# Krause corpuscles are genital vibrotactile sensors for sexual behaviours

**DOI:** 10.1038/s41586-024-07528-4

**Published:** 2024-06-19

**Authors:** Lijun Qi, Michael Iskols, Rachel S. Greenberg, Jia Yin Xiao, Annie Handler, Stephen D. Liberles, David D. Ginty

**Affiliations:** 1 Department of Neurobiology, Howard Hughes Medical Institute, Harvard Medical School, Boston, MA USA; 2 Department of Cell Biology, Howard Hughes Medical Institute, Harvard Medical School, Boston, MA USA

**Keywords:** Touch receptors, Sexual behaviour

## Abstract

Krause corpuscles, which were discovered in the 1850s, are specialized sensory structures found within the genitalia and other mucocutaneous tissues^1–4^. The physiological properties and functions of Krause corpuscles have remained unclear since their discovery. Here we report the anatomical and physiological properties of Krause corpuscles of the mouse clitoris and penis and their roles in sexual behaviour. We observed a high density of Krause corpuscles in the clitoris compared with the penis. Using mouse genetic tools, we identified two distinct somatosensory neuron subtypes that innervate Krause corpuscles of both the clitoris and penis and project to a unique sensory terminal region of the spinal cord. In vivo electrophysiology and calcium imaging experiments showed that both Krause corpuscle afferent types are A-fibre rapid-adapting low-threshold mechanoreceptors, optimally tuned to dynamic, light-touch and mechanical vibrations (40–80 Hz) applied to the clitoris or penis. Functionally, selective optogenetic activation of Krause corpuscle afferent terminals evoked penile erection in male mice and vaginal contraction in female mice, while genetic ablation of Krause corpuscles impaired intromission and ejaculation of males and reduced sexual receptivity of females. Thus, Krause corpuscles of the clitoris and penis are highly sensitive mechanical vibration detectors that mediate sexually dimorphic mating behaviours.

## Abstract (web summary)

Krause corpuscles of the clitoris and penis are highly sensitive mechanical vibration detectors that mediate sexually dimorphic mating behaviours in mice.

## Main

Somatosensory end organs are specialized for the functions of the body region or skin type in which they reside. For example, Meissner corpuscles located in dermal papillae of glabrous skin underlie light touch perception and support fine sensory–motor exchange and dexterity of the hands and digits, while, in hairy skin, longitudinal lanceolate ending complexes associated with hair follicles mediate sensory responses to hair deflection^5^. Although we have a deep understanding of the somatosensory end organs associated with glabrous and hairy skin, the physiological properties and functions of sensory structures within the mammalian genitalia are unclear.

In the late Nineteenth century, Wilhelm Krause first described specialized sensory corpuscles located in human genitalia and other mucocutaneous tissues, including the lips, tongue and conjunctiva of the eye^2–4^. He found that corpuscles of the penis and clitoris display either a glomerular shape and contain coiled axons, or they are smaller in size, possess a cylindric shape and contain simple axonal endings. These sensory structures have been assigned a number of names, including mucocutaneous end-organs^2^, Krause corpuscles, Krause end bulbs and genital corpuscles^1,6^; here we use the name ‘Krause corpuscles’ for these sensory end organs of the male and female genitalia. Although the morphological properties of Krause corpuscles were described long ago, their physiological properties and functions have remained a subject of speculation. Here we describe the anatomical and physiological properties of Krause-corpuscle-innervating sensory neurons of the clitoris and penis and their functions in sexual behaviour.

## Distribution of Krause corpuscles in mouse genitalia

**Fig. 1:**
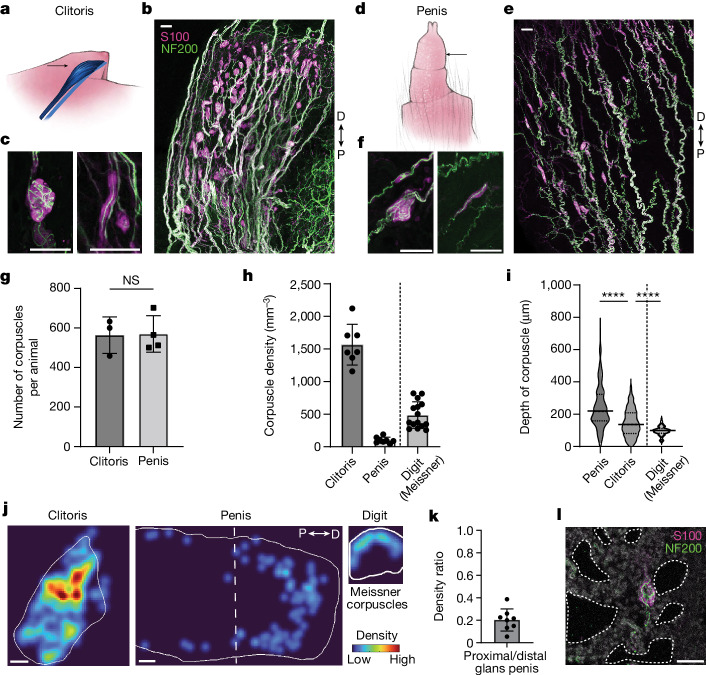
Krause corpuscles are distributed across female and male mouse genitalia. **a**, Illustration of the mouse clitoris (blue, black arrow) within the genital protrusion. **b**, Sagittal section (200 µm) of the clitoris stained for S100 and NF200. **c**, Examples of complex (left) and simple (right) Krause corpuscles in the clitoris. **d**, Illustration of the glans penis (arrow) externalized from the retracted prepuce. **e**, Sagittal section (200 µm) of the adult glans penis stained as in **b**. **f**, Examples of complex (left) and simple (right) Krause corpuscles in the penis. **g**, The total number of corpuscles summed across all sections of the clitoris or penis from each animal. Statistical analysis was performed using unpaired *t*-tests; *P* = 0.94; NS, not significant. *n* = 3 (female) and 4 (male) mice. **h**, The density of Krause corpuscles in the mouse clitoris and penis, compared with that of Meissner corpuscles in the digit (7 sections from 4 female mice; 8 sections from 2 male mice; and 15 sections from digits of 2 mice). **i**, Violin plot of the depth of corpuscles from the nearest tissue surface (*n* = 365 (penis), 554 (clitoris) and 130 (digit) corpuscles). Data are median ± quartiles. Analysis using one-way analysis of variance (ANOVA) with post hoc multiple-comparison test showed an effect of tissue type (*F*_2,1,046_ = 151.2, *P* < 0.0001); ****P < 0.0001. **j**, Example heat map of corpuscle density across a clitoris, penis and digit. Heat maps are normalized equally across the tissue types to the densest region of the clitoris. **k**, The ratio of the number of corpuscles located in the proximal half of the glans penis to that in the distal half. **l**, Example of a Krause corpuscle adjacent to expanded cavernous space (dotted lines) in a penis fixed in the erect state and stained as in **b**. Scale bars, 200 µm (**j**) and 50 µm (**b**, **c**, **e**, **f** and **l**). D, distal; P, proximal. For **g**, **h** and **k**, data are mean ± s.d. The diagrams in **a** and **d** were created by G. Park. Source Data

**Extended Data Fig. 1:**
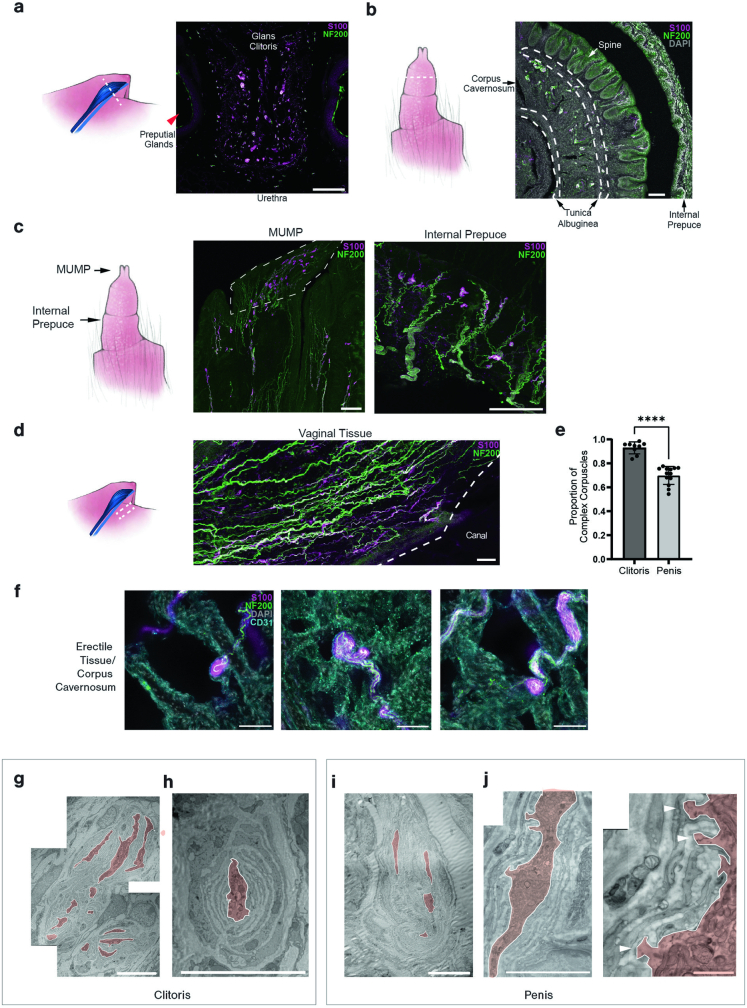
Distribution and morphological features of Krause corpuscles. **a**, Immunostaining of a coronal section through the clitoris and surrounding tissue (left dashed line) for S100 and NF200. The clitoris, urethra, and preputial glands are annotated. **b**, Immunostaining of a coronal section through the penis as in **a**. The internal prepuce, penile spines of the glans penis, corpus cavernosum, and surrounding tunica albuginea are annotated. **c**, Corpuscles within the male urogenital mating protuberance (MUMP) and internal prepuce are visualized as in **a**. **d**, Immunostaining of a section of vaginal tissue, stained as in **a**, isolated from the dashed area, shows an absence of Krause corpuscles. **e**, Quantification of the percent of complex Krause corpuscles among the total corpuscles observed in a section of genital tissue (9 sections from 4 females and 13 sections from 4 males). ****p < 0.0001, unpaired t-test. **f**, Immunostained sections showing Krause corpuscles within the corpus cavernosum of erect penis tissue for S100, NF200, and CD31. **g**, Representative transmission electron micrograph of a multi-lobulated complex Krause corpuscle within the clitoris. **h**, Example electron micrograph of a simple Krause corpuscle with a single lamellated axon profile within the clitoris. **i**, Example electron micrograph of a complex Krause corpuscle within the penis. **j**, Example electron micrograph of a Krause corpuscle (left) magnified to visualize axonal protrusions (right, white arrowheads). All axon profiles in **g**-**j** were manually outlined and filled (light red). Scale bar is 100 µm in **a**-**d**, 50 µm in **f**, 10 µm in **g**-**i**, 5 µm in **j** (left), 1 µm in **j** (right). Error bars, s.d. The diagrams in **a**–**d** were created by G. Park. Source Data

To assess the distribution and density of Krause corpuscles in the genitalia of mice, we stained thick (200 µm) sagittal sections of genital tissue for neurofilament 200 (NF200) to visualize large-calibre sensory axons and S100 for terminal Schwann cells, which wrap around sensory axon terminals to form corpuscles. In female genitalia, a very high density of Krause corpuscles was observed throughout the clitoris, which is located within the visible protrusion of hairy skin, dorsal to the distal urethra and between the preputial glands^7^ (Fig. 1a–c and Extended Data Fig. 1a). Notably, these end-organ structures were absent from vaginal tissue (Extended Data Fig. 1d). In male genitalia, corpuscles were observed throughout the glans penis (Fig. 1d–f) and the internal prepuce, which is a thin sheath covering the glans^7^ (Extended Data Fig. 1b,c). While earlier reports estimated clitoral and penile sensory neuron innervation density by measuring the number of nerve fibres entering the genitalia^8^ or using small fields of view^9,10^, we obtained a comprehensive, quantitative assessment of female and male Krause corpuscles by counting the total number of corpuscles across the entire genital tissue (Fig. 1g). Notably, despite the different sizes of the female and male genitalia, the total number of Krause corpuscles within the glans clitoris and glans penis was comparable, therefore resulting in a 15-fold higher density of Krause corpuscles in the glans clitoris than in the flaccid glans penis (Fig. 1h). For comparison to another highly sensitive skin region, the density of Meissner corpuscles in the digit tips was assessed, revealing threefold more Krause corpuscles per unit volume of the clitoris compared with the Meissner corpuscles of digit skin (Fig. 1h).

**Extended Data Fig. 2:**
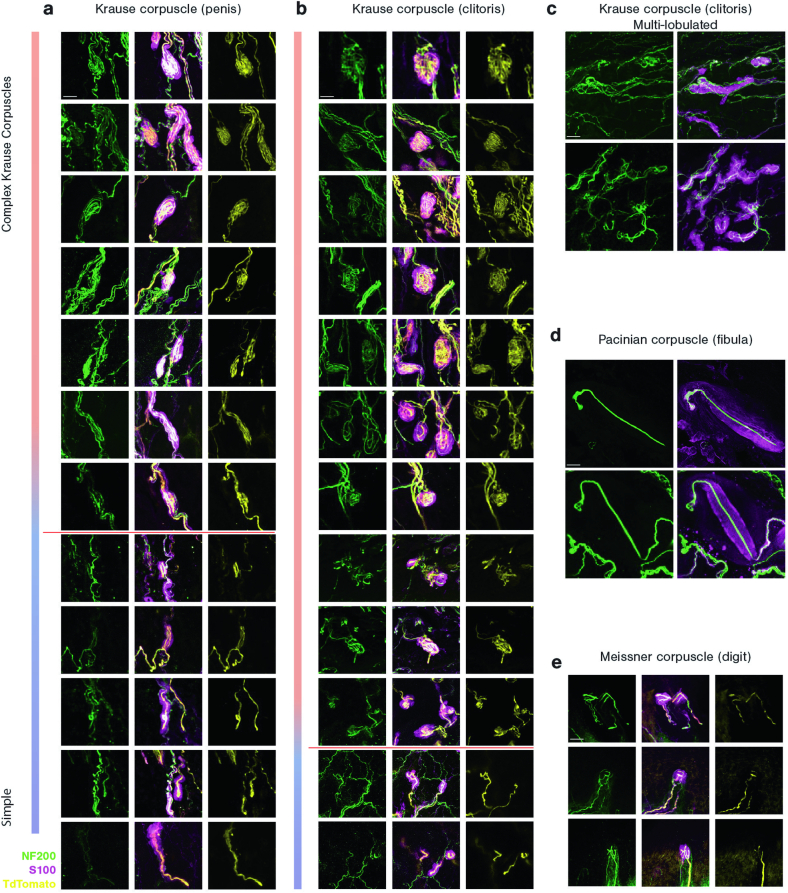
The diversity of Krause corpuscle morphologies and comparison with Meissner and Pacinian corpuscles. The range of Krause corpuscle morphologies found in the penis **a** and clitoris **b**, from complex (top) to simple (bottom). The red line depicts the distinction between complex (above the line) and simple (below the line) Krause corpuscles for quantification in Fig. 1e. **c**, Examples of multi-lobulated Krause corpuscles observed in the clitoris. **d**, Examples of Pacinian corpuscles located in the periosteum of the fibula. **e**, Examples of Meissner corpuscles located in the glabrous dermal papilla of a forepaw digit tip. The scale bar is 20 µm, and all images are of the same scale. Images in **a**, **b** and **e** are stained for NF200 (green), S100 (magenta), and tdTomato for TrkB axons (yellow) in *TrkB*^*CreER*^*; Avil*^*FlpO*^*; R26*^*FSF-LSL-Tdtomato*^ mice. Images in **c** and **d** are stained for NF200 (green) and S100 (magenta).

Morphologically, Krause corpuscles of the mouse genitalia spanned a range of shapes and sizes (Fig. 1c,f and Extended Data Fig. 2a,b). At one end of this range, corpuscles contained multiple axons tightly coiled like balls of yarn, resembling Krause’s glomerular corpuscles or end-bulbs, while, at the other end, corpuscles consisted of one or two linear axons, resembling Krause’s ‘cylindrical’, ‘simple lamellar’ or ‘Golgi–Mazzoni’ corpuscles^1^. We refer to the former as complex Krause corpuscles and the latter as simple Krause corpuscles. A greater proportion of complex Krause corpuscles was observed in clitoral tissue (93 ± 5% of corpuscles) compared with penile tissue (70 ± 7%) (Extended Data Fig. 1e). Moreover, the clitoris uniquely contained a population of elaborate, lobulated Krause corpuscles with multiple clusters of S100^+^ cells (Extended Data Fig. 2c). Despite a range in the number of axon profiles and terminal complexity across the simple and complex Krause corpuscles, electron microscopy analysis showed that axons in both types of corpuscle were concentrically wrapped by lamellar processes (Extended Data Fig. 1g–j), as previously described for Krause corpuscles in the rat^11^ and human^9^ genitalia.

In contrast to Meissner corpuscles, which strictly reside within dermal papillae of glabrous skin, Krause corpuscles were broadly distributed across both the clitoris and penis (Fig. 1j), often forming along axon bundles with NF200^+^ fibres on opposing sides of the structure (Fig. 1c,f and Extended Data Fig. 2a,b). In the penis, an enrichment of corpuscles within the erectile tissue (corpus cavernosum) and in the distal region was also observed (Fig. 1j,k). In fact, penile tissue prepared in the erect state displayed corpuscles directly adjacent to expanded cavernous spaces^11^, lined by CD31^+^ endothelial cells (Fig. 1l and Extended Data Fig. 1f), potentially rendering them responsive to vascular engorgement or pressure changes during erection^12^. Together, these findings establish the presence of morphologically diverse Krause corpuscles within the mouse genitalia, the structural similarity between mouse and human Krause corpuscles and the extremely high density of complex Krause corpuscles within the clitoris.

## DRG neurons innervating Krause corpuscles

**Fig. 2:**
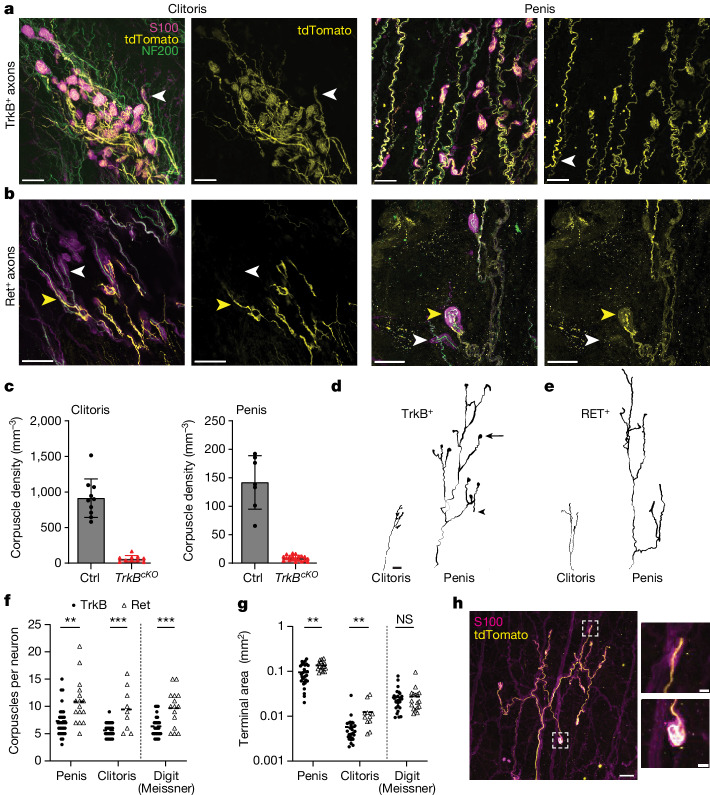
Krause corpuscles are innervated by TrkB^+^ and Ret^+^ afferents with sexually dimorphic terminal fields. **a**, Representative images of Krause corpuscles in the clitoris and penis labelled in *TrkB*^*creER*^*;Avil*^*FlpO*^*;R26*^*FSF-LSL-Tdtomato*^ mice treated with tamoxifen (TAM) at P5 (simple corpuscles, white arrowheads). **b**, Examples of Krause corpuscle afferents labelled in *Ret*^*creER*^*;Avil*^*FlpO*^*;R26*^*FSF-LSL-Tdtomato*^ mice treated with tamoxifen at E11.5 or E12.5 (complex corpuscles, yellow arrowheads; simple corpuscles, white arrowheads). **c**, The density of corpuscles in the clitoris and penis of *TrkB*^*flox/flox*^ (Ctrl) and *Avil*^*cre*^*;TrkB*^*flox/flox*^ (*TrkB*^*cKO*^) mice (*n* values are shown in the Methods). **d**,**e**, Reconstructed single axons in the clitoris and penis labelled in *TrkB*^*creER*^*;Brn3a*^*cKOAP*^ (**d**) and *Ret*^*creER*^*;Brn3a*^*cKOAP*^ (**e**) mice. The AP signals in a single TrkB^+^ axon often spread into both bulbous (arrow) and linear endings (arrowhead). Scale bar, 50 µm. **f**, The number of Krause corpuscles innervated by single TrkB^+^ or Ret^+^ afferents in the penis and clitoris, or Meissner corpuscles in digits of the paw, observed with sparse AP labelling (*n* values are provided in the Methods). Analysis using two-way ANOVA revealed no effect of tissue type within TrkB^+^ or Ret^+^ neurons (*F*_1,116_ = 2.9, *P* = 0.059); comparisons between TrkB^+^ and Ret^+^ neurons of each tissue type were performed using multiple unpaired *t*-tests; **P < 0.01, ***P < 0.001. Black bars represent the mean. **g**, The area encompassed by the terminals of individual TrkB^+^ or Ret^+^ afferents of the penis, clitoris and digit, plotted on a log_10_ scale. Statistical analysis was performed using multiple unpaired *t*-tests. Data were obtained from the same number of sections and animals as in **f**. **h**, Example of a single TrkB^+^ axon, sparsely labelled in a *TrkB*^*creER*^*;Avil*^*FlpO*^*;R26*^*FSF-LSL-Tdtomato*^ mouse, terminating in both simple (top) and complex (bottom) Krause corpuscles. Scale bars, 50 µm (**a**, **b** and **h** (main image)) and 10 µm (**h** (inset)). For **c**, data are mean ± s.d. Source Data

**Extended Data Fig. 3:**
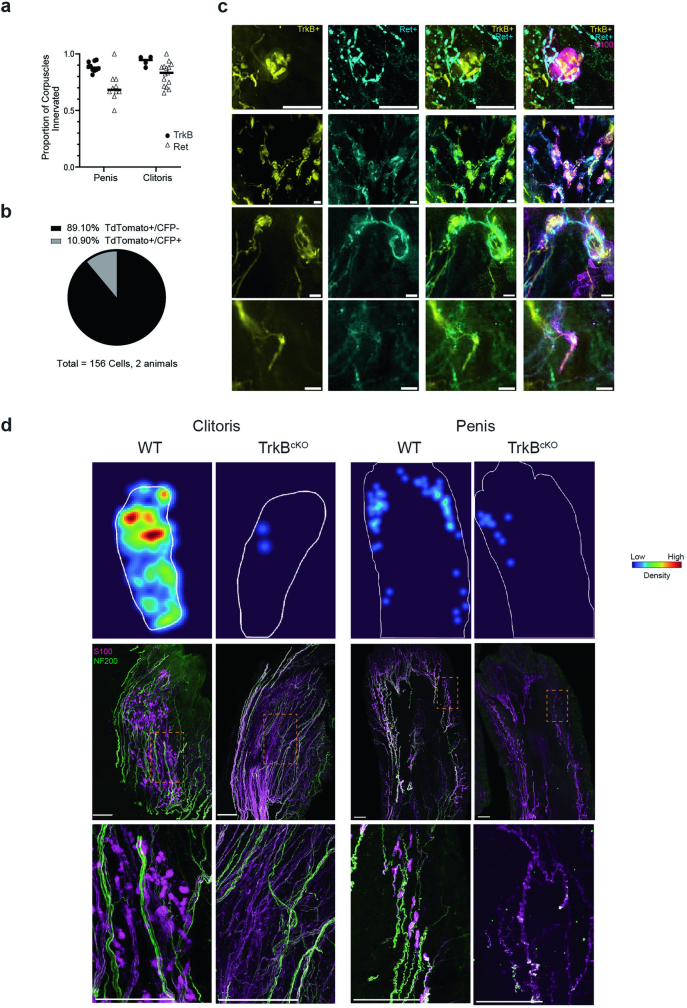
Krause corpuscles are dually innervated by TrkB^+^ and Ret^+^ fibres, and *TrkB* is required for formation of Krause corpuscles. **a**, Quantification of the fraction of corpuscles innervated by NF200^+^ axons labelled using *TrkB*^*CreER*^*; Avil*^*FlpO*^*; R26*^*FSF-LSL-Tdtomato*^ or *Ret*^*CFP*^ mice, among all S100^+^ corpuscles in a given section (TrkB: 10 sections from three males and four sections from two females; Ret: 10 sections from two males and 15 sections from three females). **b**, Quantification of the overlap of TdTomato and CFP in DRG soma of *TrkB*^*CreER*^; Ai14; *Ret*^*CFP*^ mice. **c**, Dual labelling of Ret^+^ and TrkB^+^ Krause corpuscle afferents showing dually innervated corpuscles and a simple corpuscle innervated only by a TrkB^+^ fibre (bottom) stained for tdTomato (TrkB^+^), CFP (Ret^+^), and S100. Scale bar is 10 µm in the first row and 50 µm in all other rows. **d**, Representative density heatmaps and immunofluorescence images of 200 µm-thick sections of the clitoris and glans penis of wild type and *TrkB*^*cKO*^ animals stained with NF200 and S100. In density heatmaps, the border of the section is outlined in white. The same colour scale, normalized by the densest area of the clitoris, is used for all four heatmaps. The regions indicated by rectangles in the immunofluorescence images are magnified in the bottom. Similar results were observed in more than 3 animals for each condition. Scale bar is 200 µm in **d**. Source Data

The physiological properties and functions of Krause corpuscles remain unclear despite their discovery over 160 years ago^4^. We therefore sought mouse genetic tools that enable in-depth morphological analysis, targeted physiological recordings and functional investigation of Krause corpuscle neurons. An initial survey of mouse genetic tools revealed that two alleles, *TrkB*^*creER*^ (also known as *Ntrk2*) and *Ret*^*creER*^^13,14^, efficiently labelled NF200^+^ Krause corpuscle neurons with high specificity in both female and male genitalia. *TrkB*^*creER*^ (tamoxifen treatment at postnatal day 5 (P5)) labelled dorsal root ganglion (DRG) sensory neuron axons that terminated in nearly all Krause corpuscles (>90%) of both the clitoris and penis (Fig. 2a and Extended Data Figs. 2a,b and 3a), and it did not label axonal endings in genital tissue other than those within Krause corpuscles. These TrkB^+^ axons formed both coiled terminals within complex Krause corpuscles and linear terminals within singly innervated, simple Krause corpuscles (Extended Data Fig. 2a,b). By contrast, Ret^+^ DRG neuron axons, labelled using the *Ret*^*c*^^*reER*^ allele (tamoxifen at embryonic day 11.5 (E11.5) or E12.5) or the *Ret*^*CFP*^ allele combined with NF200 staining, innervated most Krause corpuscles (around 70–80%) and were accompanied by additional Ret^−^NF200^+^ axons (Fig. 2b and Extended Data Fig. 3a). These findings raised the possibility that complex Krause corpuscles are dually innervated by TrkB^+^ and Ret^+^ DRG neurons. To directly test this, we used *TrkB*^*creER*^*;R26*^*LSL-tdTomato*^*;Ret*^*CFP*^ mice to simultaneously visualize axonal endings of the TrkB^+^ and Ret^+^ DRG neuron populations, revealing that they are two distinct subtypes (Extended Data Fig. 3b). Using this approach, we estimated that around 70% of Krause corpuscles are innervated by both TrkB^+^ and Ret^+^ fibres. These double-labelling experiments showed that complex Krause corpuscles contained extensively coiled TrkB^+^ axons and less branched, more peripherally localized Ret^+^ axons, while simple Krause corpuscles contained linear TrkB^+^ axons but lacked Ret^+^ axons (Fig. 2a,b and Extended Data Fig. 3c). While this dual-innervation pattern of Krause corpuscles is reminiscent of Meissner corpuscles in glabrous skin^15^, Krause corpuscles exhibited distinct axonal coiling and distribution patterns (Fig. 1i–k and Extended Data Fig. 2). Also similar to Meissner corpuscles^15^, TrkB signalling in DRG sensory neurons is essential for Krause corpuscle formation, as Krause corpuscles were nearly absent in both the clitoris and penis of mice lacking TrkB in sensory neurons (*Avil*^*cre*^*;TrkB*^*flox/flox*^ mice, referred to as *TrkB*^*cKO*^ mice) (Fig. 2c and Extended Data Fig. 3d).

We also visualized axonal arborization patterns of individual TrkB^+^ and Ret^+^ Krause corpuscle afferents using sparse genetic labelling and whole-mount alkaline phosphatase (AP) staining of genital tissue (Fig. 2d,e). In both the clitoris and the penis, individual Ret^+^ DRG neurons innervated a greater number of corpuscles and covered a larger terminal area compared with TrkB^+^ neurons (Fig. 2f,g). Furthermore, the terminal innervation areas of individual TrkB^+^ and Ret^+^ DRG neurons were 11 and 16 times smaller, respectively, in the clitoris compared with the penis (Fig. 2g), despite these neurons forming a similar number of corpuscles (Fig. 2f). This finding is aligned with the 15-fold higher density of Krause corpuscles observed in the clitoris compared with the penis (Fig. 1h). Moreover, we observed that the terminals formed by an individual TrkB^+^ neuron may include both bulbous and linear endings (Fig. 2d,h), indicating that a single TrkB^+^ neuron can innervate both types of Krause corpuscle. This diversity of terminal structures associated with individual Krause corpuscle afferents may endow them with a range of sensitivities or tuning properties.

**Extended Data Fig. 4:**
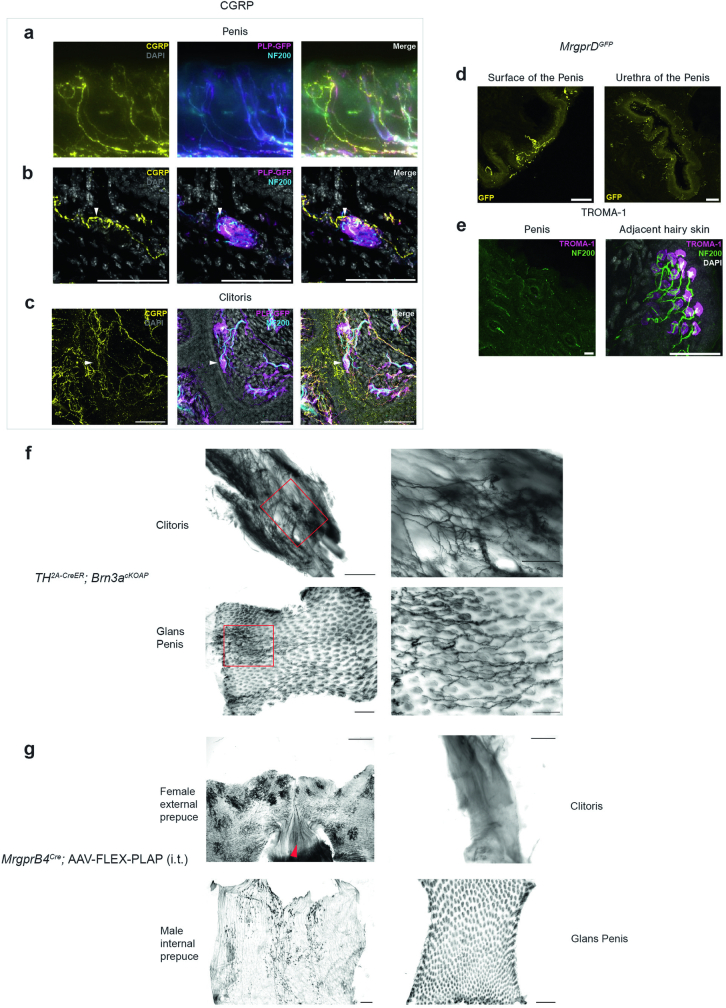
Glans clitoris and penis are innervated by CGRP^+^, MrgprD^+^, and TH^+^, but not MrgprB4^+^, fibres. **a**-**c**, Representative images of immunostained glans penis sections (200 µm thick) (superficial tissue in **a**, deeper tissue in **b**) and clitoris sections (200 µm thick, **c**) for CGRP, NF200, and GFP to label Schwann cells in *PLP*^*EGFP*^ animals. The white arrowheads in **b** and **c** indicate Krause corpuscles that have CGRP^+^ fibre passing through or terminating within them. **d**, GFP immunostaining of coronal sections of the glans penis from *MrgprD*^*GFP*^ animals. Left: superficial free nerve endings; right: nerve terminals near the urethra of the penis. **e**, Representative images of co-staining of TROMA-1, a marker of Merkel cells, and NF200. Left: a section of glans penis; right: a section of adjacent hairy skin. **f**, Whole-mount AP staining of the mouse clitoris (top) and glans penis (bottom) from *Th*^*2a-CreER*^*;Brn3a*^*cKOAP*^ mice, treated with tamoxifen at 3 weeks old. *Brn3a*^*cKOAP*^ reporter line does not label TH^+^ sympathetic fibres. The areas within red rectangles in the left images are shown at higher magnification in the right images. **g**, Whole mount AP staining of MrgprB4^+^ fibres in hairy skin (external prepuce, top left) surrounding the genital region, isolated clitoris (top right), internal prepuce (bottom left), and glans penis (bottom right). The red arrow in the top left image indicates the vaginal opening connected to the adjacent hairy skin. Similar results were observed in more than 3 animals for each condition. Scale bar is 50 µm in **a**-**e**, 500 um in the left images of **f** and in all four images in **g**, 200 um in the right images of **f**.

In addition to Krause-corpuscle-associated neurons, we observed free nerve endings formed by other DRG sensory neuron subtypes in the genitalia, including CGRP^+^ fibres, MRGPRD^+^ fibres and NF200^+^ fibres, that are not corpuscle associated. These free nerve endings were observed throughout the genital tissue, often terminated close to the surface of the tissue, and emerged from axons that occasionally passed through Krause corpuscles (Extended Data Fig. 4a–d). TH^+^ sensory neurons, which in hairy skin are C-fibre low-threshold mechanoreceptors (C-LTMRs)^16^, also innervated the glans clitoris and penis (Extended Data Fig. 4f). Moreover, we found that MRGPRB4^+^ fibres innervated the prepuce but not the glans clitoris or penis (Extended Data Fig. 4g). Notably, Merkel cells, which associate with slowly adapting low-threshold mechanoreceptors^17^, were absent from genital tissue, although they were observed in abundance in adjacent hairy skin (Extended Data Fig. 4e). Thus, while several DRG neuron subtypes innervate the genitalia, TrkB^+^ and Ret^+^ DRG sensory neurons uniquely form Krause corpuscles.

## Central projections of Krause corpuscle afferents

**Fig. 3:**
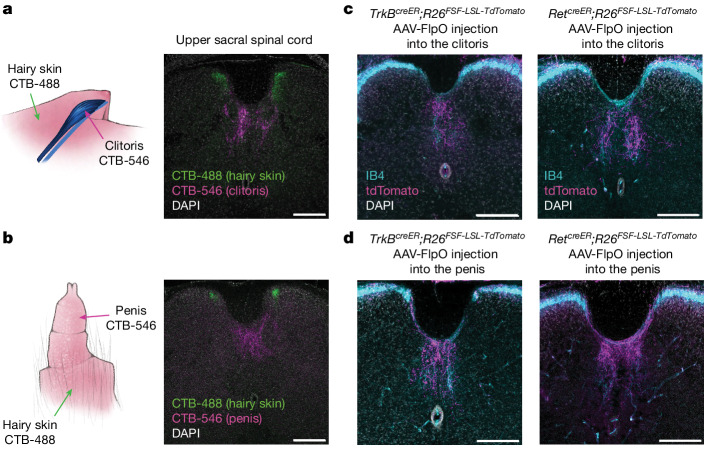
Krause corpuscle afferents project to the dorsomedial spinal cord. **a**,**b**, Retrograde labelling of DRG neurons innervating the clitoris (**a**) or penis (**b**) and the adjacent midline hairy skin region using CTB tracers conjugated to different fluorophores (hairy: CTB-488; genital: CTB-546). Coronal sections of the upper sacral spinal cord are shown on the right. **c**,**d** Labelling of Krause corpuscle afferents using injection of AAV2-retro-hSyn-FlpO into the clitoris (**c**) or glans penis (**d**) of *TrkB*^*creER*^*;R26*^*FSF-LSL-Tdtomato*^ (tamoxifen, 0.5 mg at P5) or *Ret*^*creER*^*;R26*^*FSF-LSL-TdTomato*^ (tamoxifen, 3 mg at E12.5) mice, with IB4 staining to label the lamina II of the spinal cord as a reference for laminar location. Similar results were observed in more than three animals per condition. For **a**–**d**, scale bars, 200 µm. The diagrams in **a** and **b** were created by G. Park.

**Extended Data Fig. 5:**
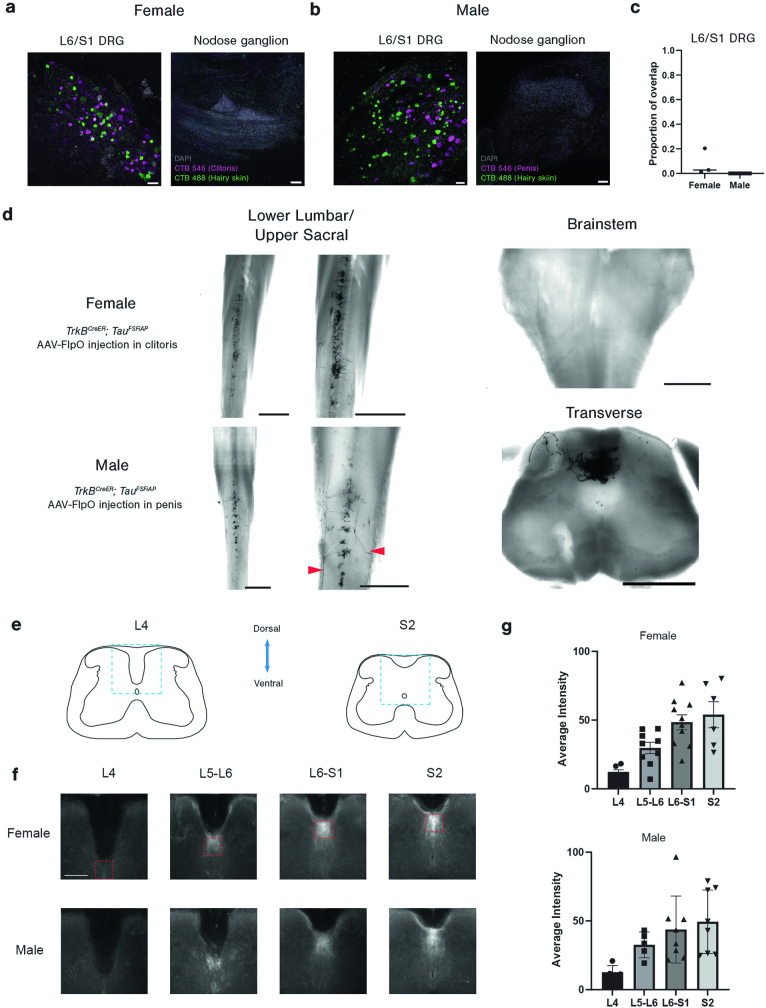
The central terminals of Krause corpuscle afferents. **a-b**, Representative images of whole-mount L6 or S1 DRGs or nodose ganglion after injection of CTB tracers conjugated to different fluorophores into genital tissues (CTB-546) and adjacent mid-line hairy skin (CTB-488). Scale bar is 50 µm. **c**, Quantification of overlapping labelling of neurons identified by injection into genitalia and hairy skin, as in **a** and **b**. **d**, Whole-mount AP staining of the spinal cord of *TrkB*^*CreER*^*; Tau*^*FSFiAP*^ animals with AAV2-retro-hSyn-FlpO injected into the clitoris (upper panels) or penis (lower panels). The middle panels show magnified images of those shown on the left. Red arrowheads point to the individual axons entering the spinal cord. The images on the right show whole mount AP staining of the female dorsal column nucleus (top) and a transverse section of the male S1 spinal cord (bottom). Scale bar in **d** is 1 mm for whole mount images and 500 µm for transverse sections of AP staining. **e**, Schematics of spinal cord sections in L4 and S2 regions, with the dashed lines indicating the region subjected to image averaging in **e**. The diagram was created by G. Park. **f**, Averaged images of spinal cord terminals of Krause corpuscle afferents, labelled by injection of AAV2-retro-hSyn-FlpO into the genitalia of *TrkB*^*CreER*^*; R26*^*FSF-LSL-Tdtomato*^ or *Ret*^*CreER*^*; R26*^*FSF-LSL-Tdtomato*^. All images are of the same size and share the common scale bar (200 μm). The red squares refer to the region selected for fluorescence intensity quantification in **g**. **g**, Quantifications of fluorescence intensity in the square region (150×150 μm) immediately ventral to the dorsal column. The number of images analysed for females is 5, 9, 10, 6, for L4, L5-L6, L6-S1, S2, respectively. For males, the corresponding numbers were 5, 5, 8, and 8. Images for both females and males were obtained from more than 3 animals per sex. Error bars, s.d.

We next examined the central termination patterns of the TrkB^+^ and Ret^+^ DRG neurons that innervate Krause corpuscles. In initial experiments, cholera toxin subunit B (CTB) tracers conjugated to different fluorophores were injected into genital tissues and, for comparison, the adjacent midline hairy skin (Fig. 3a,b). This labelled anatomically distinct populations of sensory neurons of which the cell bodies reside within bilateral L6 and S1 DRGs, but not within the nodose ganglia, which innervates certain internal organs through projections of the vagus nerve^18^ (Extended Data Fig. 5a,b). CTB-labelled cell bodies of DRG neurons that innervate the genitalia and adjacent hairy skin regions were almost entirely non-overlapping (Extended Data Fig. 5c). Notably, the central terminals of sensory neurons innervating the genital tissue and adjacent hairy skin were observed in the lower lumbar and upper sacral spinal cord in highly segregated patterns (Fig. 3a,b). While sensory neurons innervating midline hairy skin terminated within medial regions of both the left and right spinal cord dorsal horn, genital-innervating sensory neurons terminated in a unique, midline region of the spinal cord, located between the dorsal column and central canal; this region is often called the dorsal grey commissure (DGC)^19,20^.

To specifically visualize the spinal cord termination patterns of TrkB^+^ and Ret^+^ Krause corpuscle afferents, we injected AAV-FlpO into the genital tissue of *TrkB*^*creER*^ or *Ret*^*creER*^ mice containing either a dual-recombinase-dependent fluorescent reporter allele (*R26*^*FSF-LSL-tdTomato*^) or a dual-recombinase-dependent AP reporter allele (*Tau*^*FSF-iAP*^). As observed in the CTB pan-neuronal labelling experiments, TrkB^+^ and Ret^+^ afferents innervating the clitoris and penis terminated exclusively in the DGC region of the spinal cord (Fig. 3c,d and Extended Data Fig. 5d). Moreover, whole-mount AP labelling experiments revealed that axons of individual TrkB^+^ Krause corpuscle afferents bifurcated after entering the spinal cord and formed clusters of collaterals extending along the rostrocaudal axis (Extended Data Fig. 5d). While collaterals were enriched between L6 and S2, some collaterals containing fewer terminal branches extended a few segments farther but did not reach the upper lumbar spinal segments or the brainstem (Extended Data Fig. 5e–g). Thus, both male and female Krause corpuscle afferents form an elaborate pattern of axonal branches that terminate within the DGC region of the lumbosacral spinal cord.

## Krause corpuscles are vibrotactile sensors

**Fig. 4:**
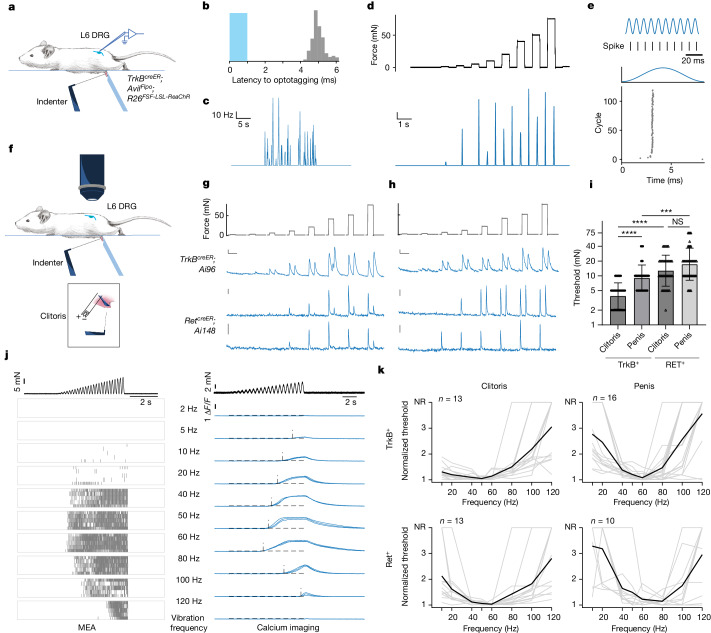
TrkB^+^ and Ret^+^ Krause corpuscle afferents are fast-conducting, low-threshold mechanical vibration sensors. **a**, Schematic of mechanical stimulation of the glans penis during in vivo MEA recording of L6 DRG neurons. The blue object on the opposite side of the indenter stabilizes the glans penis. **b**, The histogram of spike latency (grey bars) after optogenetic activation (1 ms pulse, blue bar) of a TrkB^+^ Krause corpuscle neuron of the penis. **c**–**e**, The optotagged TrkB^+^ neuron in **b** exhibits robust activation by brushing (**c**), rapid adaptation to step indentations (**d**) and phase-locking properties to sinewave vibration stimuli (120 Hz, 19 mN) (**e**). **f**, Schematic of in vivo calcium imaging of L6 DRG neurons. Inset: electrical stimulation was applied to the exposed dorsal nerve of clitoris after mechanical stimulation of the clitoris. **g**,**h**, Representative calcium signals after step indentations in the clitoris (**g**) and penis (**h**). For **g** and **h**, scale bars, 5% Δ*F*/*F* (vertical) and 5 s (horizontal). All TrkB^+^ neurons (13 in the clitoris, 16 in the penis) and a subset of Ret^+^ neurons (9 out of 18 in the clitoris, 8 out of 10 in the penis) showed an ON–OFF response, while the remaining Ret^+^ neurons showed only an ON response (bottom). **i**, Comparison of indentation thresholds (log_2_ scale) of TrkB^+^ and Ret^+^ Krause afferents. Statistical analysis was performed using two-way ANOVA with Tukey’s multiple-comparison test. **j**, Representative responses of TrkB^+^ Krause corpuscle afferents to ramping-force vibration stimuli of varying frequencies applied on the penis (top). Left, raster plot of spikes from an optotagged TrkB^+^ neuron across five repeated trials. Right, calcium signals from a TrkB^+^ Krause afferent across three trials. The horizontal dashed lines indicate the criteria of response (5 × s.d. of the baseline); the vertical dashed lines indicate the timing of response onset. **k**, Normalized frequency turning curves. The mechanical threshold for each vibration frequency is normalized to the minimum threshold of the neuron (individual neurons, grey; average, black). NR, no response. *n* indicates the number of neurons. For **i**, data are mean ± s.d. The diagrams in **a** and **f** were created by G. Park. Source Data

**Extended Data Fig. 6:**
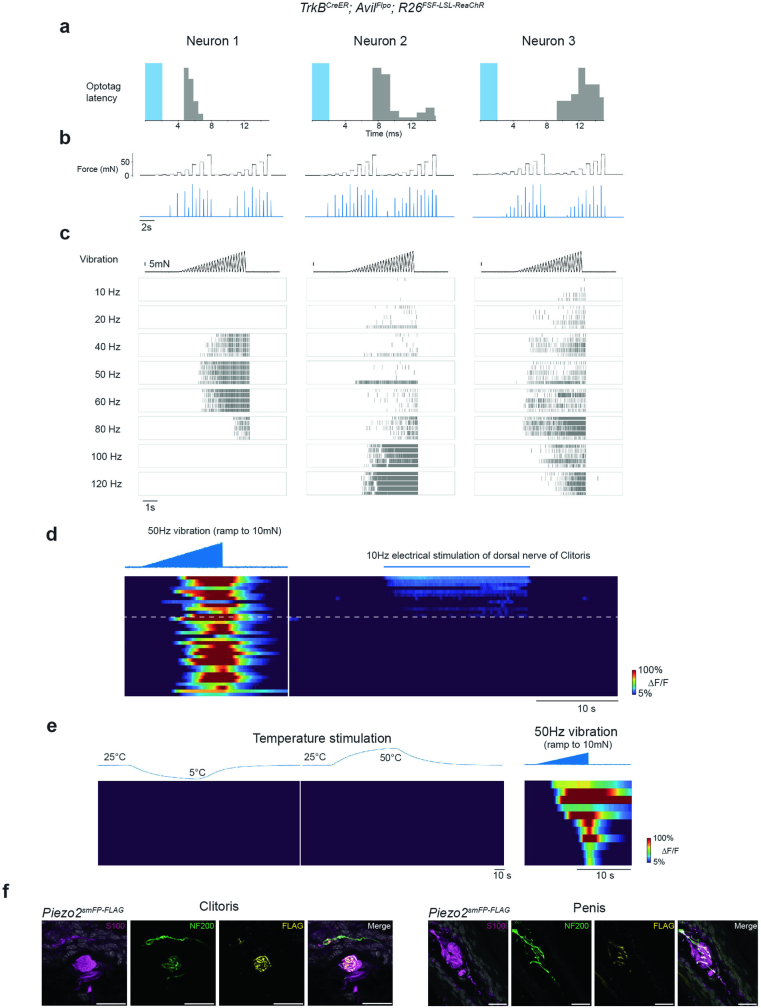
Krause corpuscle afferents are mechanosensitive neurons tuned to dynamic, light touch and mechanical vibration. **a**-**c**, Additional examples of optotagged TrkB^+^ Krause corpuscle afferents. **a**, Latency of spikes recorded in the DRG after optogenetic activation (2 ms pulse, shown in blue) of neurons labelled in *TrkB*^*CreER*^*; Avil*^*Flpo*^*; R26*^*FSF-LSL-ReaChR*^ male mice. **b**, Firing rates in response to step indentation of the glans penis. **c**, Raster plots of the response to ramping vibratory stimuli (example shown on top) of different frequencies from five repeated trials. **d**, Calcium signals of TrkB^+^ neurons in L6 DRG in response to ramping vibration stimuli applied to the protrusion where the clitoris is located (left) and to electrical stimulation of the dorsal nerve of the clitoris (right). Neurons responding to the electrical stimulation were identified as clitoris-innervating neurons (above the dashed line). The neurons below the dashed line are presumed to innervate the adjacent hairy skin. The neurons shown in **d** are from three female mice. **e**, Left: thermal responses of TrkB^+^ afferents that innervate the penis. The top trace shows the measured temperature of the water bath in which the penis was submerged. Right: the response of the same group of neurons to vibration applied to the glans penis. **f**, Representative images of Piezo2 expression in the axons innervating Krause corpuscles from *Piezo2*^*smFP-FLAG*^ animals. Similar results were observed in more than 3 animals for each condition. Sections were immunostained for S100, NF200, and FLAG. Scale bar: 20 µm.

Genetic access to Krause corpuscle afferents enabled us to address long-standing questions of their physiological properties and functions. We therefore developed a preparation for direct mechanical and thermal stimulation of the external genitalia during in vivo electrophysiological recording and calcium imaging of TrkB^+^ and Ret^+^ Krause-corpuscle-innervating neurons within L6 DRGs. Using in vivo multielectrode array (MEA) recordings of L6 DRG neurons, TrkB^+^ Krause corpuscle afferents were optotagged^21^ using *TrkB*^*creER*^;*Avil*^*Flpo*^;*R26*^*FSF-LSL-ReaChR*^ mice (Fig. 4a,b). In male mice, TrkB^+^ Krause corpuscle neurons were activated using a combination of mechanical and optogenetic stimuli applied to the glans penis to establish their receptive field locations. Male TrkB^+^ Krause corpuscle afferents exhibited conduction velocities characteristic of A fibres^5^ (*n* = 4; Extended Data Fig. 6a) and robust activation by light mechanical stimulation of the penis (Fig. 4b,c). These neurons exhibited low mechanical thresholds (1–10 mN), rapid adaptation (RA) to step indentations, firing at the onset and offset but not the sustained phase of indentations (Fig. 4d and Extended Data Fig. 6b), and precise phase locking to each cycle of mechanical vibrations up to 120 Hz, the highest frequency tested (Fig. 4e).

Owing to the low throughput of the electrophysiological recordings, we complemented the electrophysiological analyses with in vivo calcium imaging experiments using both male and female mice that express GCaMP6 in the TrkB^+^ or Ret^+^ DRG sensory neuron populations (Fig. 4f). Precise mechanical stimuli applied to the glans penis allowed identification of TrkB^+^ or Ret^+^ Krause corpuscle afferents of male mice, while electrical stimulation of the dorsal nerve of the clitoris^7^ was used to distinguish between clitoris-innervating afferents and neurons that innervate the overlying hairy skin of female mice (Extended Data Fig. 6d). Consistent with the findings from the in vivo MEA electrophysiological recordings in the male mice, these calcium imaging experiments revealed that both penis- and clitoris-innervating TrkB^+^ neurons are RA-LTMRs, exhibiting both an ON and an OFF response to step indentations of the genitals (Fig. 4g,h). This analysis also showed that TrkB^+^ sensory neurons innervating the clitoris are more sensitive than those innervating the penis (Fig. 4i). Moreover, as a population, the Ret^+^ Krause corpuscle afferents in both the penis and clitoris exhibited higher mechanical force thresholds than the TrkB^+^ afferents, and a subset of Ret^+^ neurons exhibited responses only to the onset of indentation (Fig. 4g–i). The distinct mechanical thresholds and response properties of TrkB^+^ and Ret^+^ Krause afferents may reflect the observed differences in their terminal morphology within corpuscles (Fig. 2) and may increase the dynamic range of forces encoded by Krause corpuscles.

**Extended Data Fig. 7:**
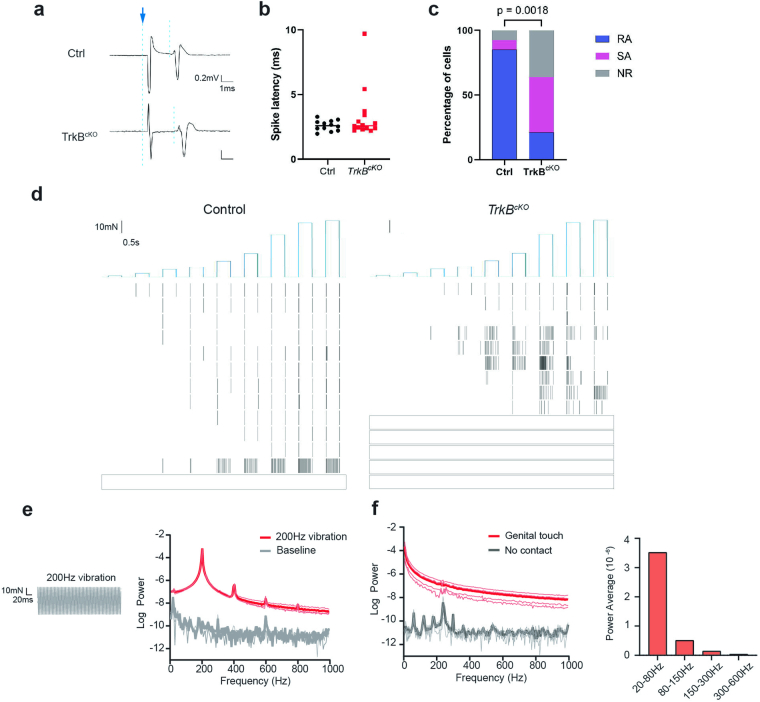
Fewer rapidly adapting penis-innervating neurons in *TrkB*^*cKO*^ mice and power spectra of vibration and genital contact. **a**, Representative traces of extracellular spikes elicited by electrical stimulation of the dorsal nerve of the penis in control (top) and *TrkB*^*cKO*^ (bottom) males. The blue arrow indicates the artifact of the electrical stimulus; the two dashed lines indicate the onset of electrical stimulation and the onset of the spike, respectively. **b**, Summary of spike latencies of genital-innervating DRG neurons recorded in control and *TrkB*^*cKO*^ males. **c**, Summary of adaption properties to step indentations of genital-innervating DRG neurons in control and *TrkB*^*cKO*^ males. Fisher’s exact test was used to test the ratio of RA neurons between controls and *TrkB*^*cKO*^ males. RA: rapid adaptation. SA: slow adaptation. NR: non-responding (to step indentation). **d**, Raster plots of all units responding to step indentations (top) in control and *TrkB*^*cKO*^ males. The most sensitive trace for each neuron is shown; a blank rectangle indicates non-responsive cells. Scale bar is 1 ms (x axis) and 0.2 mV (y axis) in **a**, 0.5 s (x axis) and 10mN (y axis) in **d**. N = 13 units for control (from 8 mice), 14 units for *TrkB*^*cKO*^ (from 10 mice) for **b**-**d**. **e**, Force measured (left) and power spectrum (right) during 200 Hz vibration applied to the force sensor. **f**, Power spectrum (left) and averaged power (right) of force generated while the isolated mouse penis was moved across vaginal tissue mounted on the force sensor. In power spectra shown in **e** and **f**, thick lines indicate mean values, while thin lines depict individual traces.

We also assessed the physiological properties of genital afferents in *TrkB*^*cKO*^ male mice, which lack Krause corpuscles (Fig. 2c), and their littermate controls. Although the alleles used to excise *TrkB* in *TrkB*^*cKO*^ mice precluded genetic labelling of TrkB^+^ and Ret^+^ afferents in these mutants, we could identify A-fibre genital afferents using electrical stimulation of the dorsal nerve of the penis during in vivo extracellular recordings in the L6 DRG (Extended Data Fig. 7a). While most A-fibre genital afferents in control mice were RA-LTMRs, as predicted^22,23^, few RA-LTMRs were observed in recordings from *TrkB*^*cKO*^ animals (Extended Data Fig. 7b,c). Thus, Krause corpuscles are required for the normal cohort of physiologically defined RA-LTMRs that innervate the genitalia.

As RA-LTMRs that innervate other body regions, including Meissner corpuscles of the digits and Pacinian corpuscles associated with the deep dermis or bones, display preferred sensitivity to certain vibration frequencies^5^, we next assessed force thresholds across a range of vibration frequencies (2–120 Hz) for the two genetically labelled Krause corpuscle afferent types. For both the clitoris and penis, TrkB^+^ and Ret^+^ Krause corpuscle neurons exhibited the lowest force thresholds in response to 40–80 Hz vibration stimuli, indicating exquisite sensitivity to vibrations in the low-frequency range (Fig. 4j,k and Extended Data Fig. 6c). As vibrations of the skin surface arise during dynamic skin–object contacts^24,25^, we also examined the range of vibrations of the genitalia that are generated during dynamic contacts between isolated penile and vaginal tissues. The power spectrum of forces measured during movement of the isolated genitalia showed a wide range of frequencies and amplitudes, with higher power observed in the low-frequency range (20–80 Hz) than in the high-frequency range (>100 Hz) (Extended Data Fig. 7e,f). These findings suggest that natural movements across the surface of genital tissues evoke vibrations within the frequency range that most effectively activates both the TrkB^+^ and Ret^+^ Krause corpuscle afferent types.

Furthermore, although early literature had proposed that Krause corpuscles are thermoreceptors based on anatomical considerations^26,27^, a notion that has persisted in contemporary texts and reviews^6,28^, we observed that TrkB^+^ Krause corpuscle afferents are insensitive to thermal stimuli (Extended Data Fig. 6e). Moreover, the mechanotransduction channel Piezo2 was highly localized to axons within, but not outside, the Krause corpuscle, and not to lamellar cells, in both the penis and clitoris (Extended Data Fig. 6f), therefore providing a molecular basis for the mechanosensitivity of Krause corpuscle afferents^29–33^. Taken together, TrkB^+^ and Ret^+^ Krause corpuscle afferents are A-fibre RA-LTMRs, exhibiting distinct mechanical thresholds and optimal sensitivity to 40–80 Hz mechanical vibrations, and TrkB^+^ Krause corpuscle afferents of the clitoris are the most sensitive of the genital innervating RA-LTMRs.

## Krause corpuscles in sexual behaviours

**Fig. 5:**
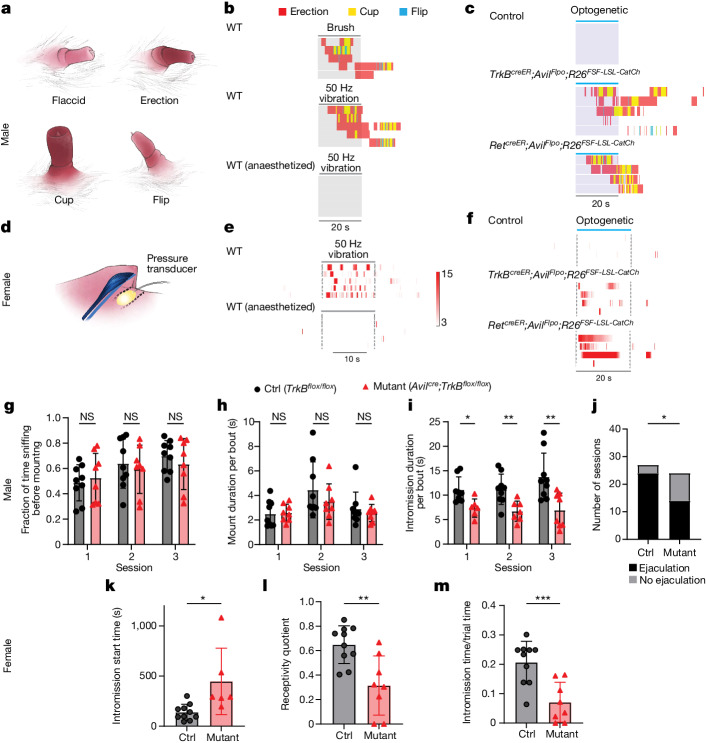
Krause corpuscles mediate normal sexual behaviours. **a**, Illustrations of the four states of the mouse penis during sexual reflexes. **b**, Ethograms depicting the reflex responses of spinalized mice to brush and 50 Hz vibration of the glans penis while awake or anaesthetized. **c**, Responses to optogenetic stimulation (20 Hz, 2 ms pulses) of the penis of mice without opsin expression, mice expressing CatCh in TrkB^+^ sensory neurons and mice expressing CatCh in Ret^+^ fibres. **d**, Illustration of the method for assessing sexual reflexes in spinalized female mice, using a balloon placed at the vaginal opening. **e**, Heat maps of vaginal pressure during 50 Hz vibration applied to the clitoris of awake or anaesthetized female mice. **f**, Vaginal pressure in response to optogenetic stimulation of the clitoris in mice of the same genotype as in **c**. The colour bar indicates the fold increase relative to the s.d. of the baseline pressure for both **e** and **f**. **g**–**i**, Comparisons of the mating behaviours of control (*n* = 9) and *TrkB*^*cKO*^ (*n* = 8) male mice: quantification of sniffing (**g**), average duration per mounting bout (**h**) and average duration per intromission bout (**i**) across three sessions that were at least 1 week apart. Statistical analysis was performed using multiple unpaired *t*-tests; *P < 0.05. **j**, Comparison of the number of sessions with successful ejaculation summed over the three sessions. Statistical analysis was performed using a Fisher’s exact test; *P* = 0.022. **k**–**m**, Comparisons of the mating behaviours of naturally cycling, experienced control (*n* = 10) and *TrkB*^*cKO*^ (*n* = 8) female mice: start time of intromission (**k**), receptivity quotient (total intromission time divided by the sum of the total intromission and mounting time) (**l**) and intromission time divided by total trial time (**m**). Statistical analysis was performed using unpaired *t*-tests. For **h**, **i** and **k**–**m**, data are mean ± s.d. The diagrams in **a** and **d** were created by G. Park. Source Data

**Extended Data Fig. 8:**
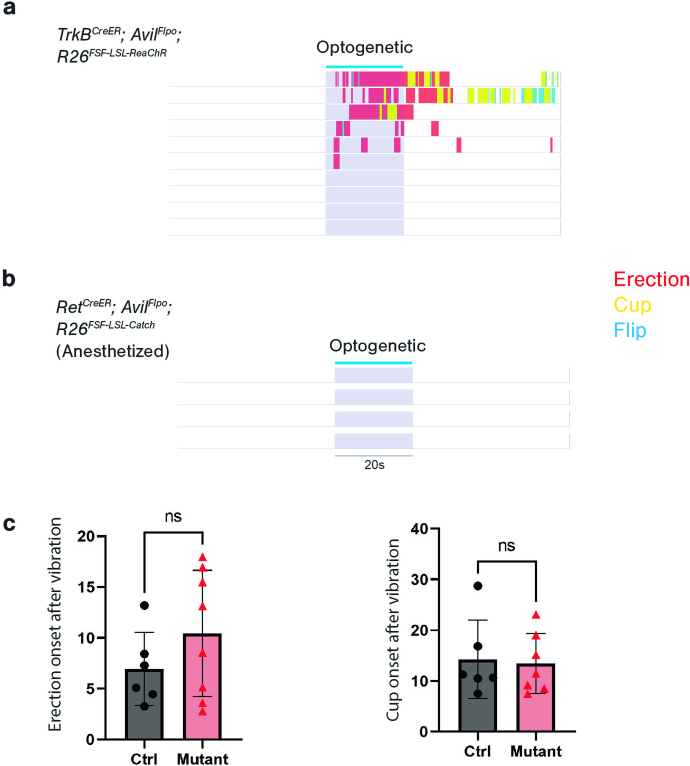
Additional experiments for sexual reflexes in male mice. **a**-**b**, Ethograms showing sexual reflex responses to optogenetic stimulation of the penis of awake mice expressing ReaChR in TrkB^+^ sensory neurons (**a**) and optogenetic stimulation of anaesthetized mice expressing CatCh in Ret^+^ fibres (**b**). **c**, Genital reflexes following 50 Hz vibration applied to the penis of control (ctrl, n = 6) and *TrkB*^*cKO*^ males (n = 8) that have undergone spinal transection. One *TrkB*^*cKO*^ animals did not exhibit a cup, thus n = 7 for the cup behaviour from *TrkB*^*cKO*^ males. Unpaired t-test, p > 0.05. Error bars, s.d.

The high density and exquisite vibrotactile sensitivity of Krause corpuscle afferents, and the availability of genetic tools to study them, prompted us to examine whether Krause corpuscles contribute to sexual behaviour. We began by testing whether the activation of Krause corpuscle afferents is sufficient to evoke sexual reflex behaviours. Previous studies have reported that restrained male mice can display mechanically evoked sexual reflexes only after spinal cord transection to eliminate descending inhibitory signals^34,35^. After spinal transection at the ninth thoracic vertebra (T9), male mice displayed robust erectile responses to brushing and 50 Hz mechanical vibration applied to the penis of awake, non-anaesthetized animals (Fig. 5a,b and Supplementary Video 1). These responses typically began with an erection, characterized by distension and reddening of the penis, often followed by the appearance of a ‘cup’, which represents flared, full engorgement with blood, and, in many cases, a ‘flip’, which is a brief dorsiflexion of the penis^34^ (Fig. 5a). As Krause corpuscle afferents were maximally activated by brush and mechanical vibrations (Fig. 4), we tested whether optogenetic activation of Krause corpuscle afferent terminals in the penis can recapitulate mechanically evoked reflex responses. We found that direct optogenetic stimulation of the penis (10 Hz, 2 ms pulse for 20 s) of *TrkB*^*creER*^*;Avil*^*Flpo*^*;R26*^*FSF-LSL-ReaChR*^ mice, which express ReaChR in TrkB^+^ Krause corpuscle afferents, led to erectile responses in 6 out of 10 animals (Extended Data Fig. 8a). Moreover, optical stimulation of the glans penis of mice expressing a faster opsin, CatCh^36^, in TrkB^+^ Krause corpuscle afferents using a higher-frequency stimulus (20 Hz, 1 ms pulse for 20 s) led to erectile responses in 5 out of 5 animals tested (Fig. 5c and Supplementary Video 2). By contrast, light pulses did not evoke erection when delivered to the penis of control animals lacking opsin expression (Fig. 5c). Moreover, optogenetic activation of Ret^+^ fibres expressing CatCh (*Ret*^*creER*^*;Avil*^*Flpo*^*;R26*^*FSF-LSL-CatCh*^, tamoxifen 3 mg at E12.5) in the glans penis also triggered the sexual reflex in awake animals (4 out of 4 mice). While Ret^+^ autonomic efferents may have been labelled with the *Ret*^*creER*^ genetic labelling strategy^37,38^, it is notable that the same optogenetic stimulation in anaesthetized animals did not evoke erectile reflexes (Extended Data Fig. 8b). As anaesthesia probably disrupts the spinal cord circuits underlying the reflex without directly blocking autonomic fibre terminals, this finding suggests that the optogenetically evoked erectile responses of awake *Ret*^*creER*^*;Avil*^*Flpo*^*;R26*^*FSF-LSL-CatCh*^ mice were probably mediated by activation of Ret^+^ Krause corpuscle afferents. Thus, activation of Krause corpuscle afferents is sufficient to initiate sexual reflexes in male mice.

We next tested mechanically and optogenetically evoked sexual reflexes of female mice. For this analysis, we adapted methods used in studies of the ‘clitorovaginal reflex’ in humans and rats^39–42^, in which mechanical stimulation of the clitoris leads to contraction of the vaginal cavity. We monitored vaginal pressure while stimulating the clitoris in awake mice after spinal transection at T9 (Fig. 5d). Mechanical vibration (50 Hz) of the clitoris elicited an increase of vaginal pressure, which was not observed in the anaesthetized state, indicating that female mice exhibit a clitorovaginal reflex (Fig. 5e). We next expressed the fast opsin CatCh in TrkB^+^ or Ret^+^ Krause corpuscle afferents and performed optogenetic stimulation of clitoral afferents by shining light onto the clitoris. Optogenetic activation of either type of Krause corpuscle afferent induced vaginal contractions in female mice (Fig. 5f), indicating that activation of Krause corpuscle afferent terminals in the clitoris is sufficient to drive sexual reflexes in female mice.

**Extended Data Fig. 9:**
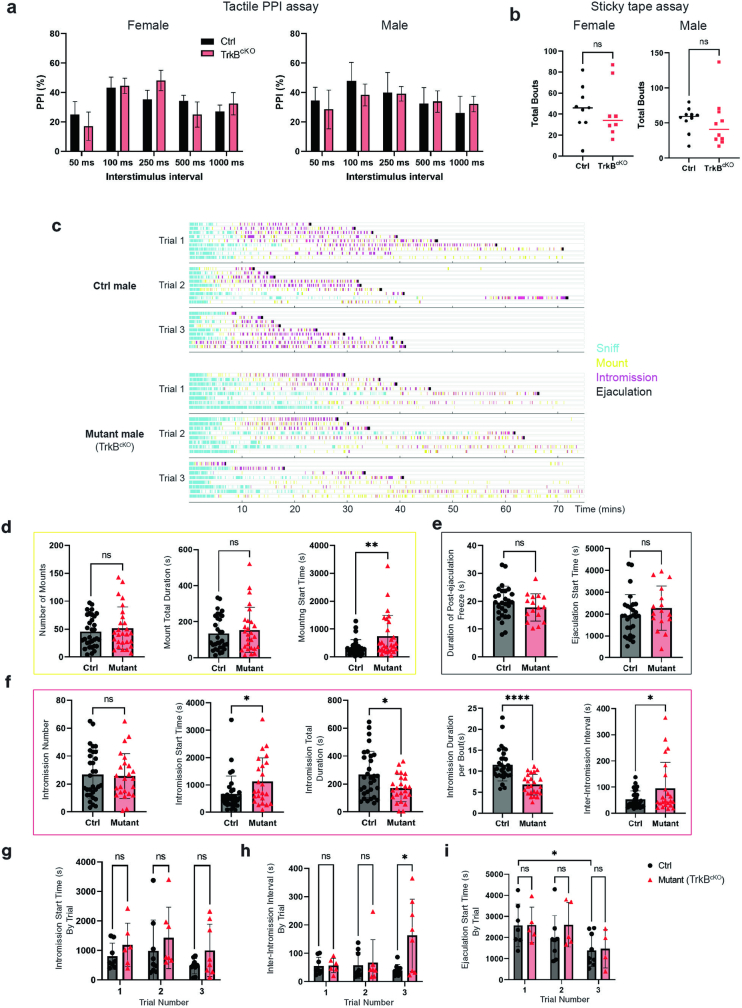
*TrkB*^*cKO*^ mice have normal hairy skin mechanosensitivity, and *TrkB*^*cKO*^ males display deficits in mating behaviours. **a**, Summary of the percent inhibition of the startle response to an acoustic stimulus (125 dB, 20 ms) caused by a light air puff (prepulse, 0.9 PSI, 50 ms) applied to the back hairy skin preceeding the acoustic stimulus at various interstimulus intervals, for both female (left) and male (right) *TrkB*^*cKO*^ mice (n = 10 for females, 8 for males) and control littermates (n = 10 for females, 8 for males; error bars refer to s.e.m.). Multiple unpaired t-test for each interstimulus interval, no significant differences for all interstimulus intervals. **b**, The total number of bouts of scratching, shaking, or biting a piece of tape stuck to back hairy skin of mice within a 5 min period, for both female (left) and male (right) *TrkB*^*cKO*^ mice (n = 8 for females, 10 for males) and control littermates (n = 9 for females, 10 for males). Unpaired t-test, p = 0.79 for females, p = 0.70 for males. **c**, Ethograms of mating behaviour of *TrkB*^*cKO*^ (n = 8) and littermate control (n = 9) males within a 75-minute testing period across three trials, with trials separated by at least one week. The sniffing bouts shown are limited to the male’s sniffing behaviour before the first mount. Mounting refers to shallow probing and postural maneuvering without gaining access to the female genitalia, while intromission represents periods of time during which long rhythmic thrusts are observed. **d**, Quantification of mounting behaviours across three trials. Unpaired t-test, ** p < 0.01. **e**, Quantification of ejaculation behaviours, collected across three trials. Only animals that achieved ejaculation are included. **f**, Quantification of intromission behaviours across three trials. Unpaired t-test, * p < 0.05, **** p < 0.0001 (for intromission start time, * p = 0.029; for intromission total duration, * p = 0.014; for inter-intromission interval, * p = 0.028). **g**-**i**, Comparison of intromission start time (**g**), inter-intromission interval (**h**), and ejaculation start time (**i**) within each trial. Unpaired t-test, * p < 0.05. All error bars from **b**-**i** refer to s.d. Source Data

To determine the necessity of Krause corpuscles in sexual behaviours, we used *TrkB*^*cKO*^ mice, which lack Krause corpuscles (Fig. 2c). We first assessed vibration-induced reflexes of control and *TrkB*^*cKO*^ spinalized male mice, finding no clear deficiency in mechanically evoked erectile responses in the mutants (Extended Data Fig. 8c). It is possible that the sexual reflex in spinalized *TrkB*^*cKO*^ animals is mediated by the few remaining RA-LTMRs in *TrkB*^*cKO*^ mice (Extended Data Fig. 7c,d), slowly adapting A fibre neurons, which are present in greater abundance in the mutants (Extended Data Fig. 7c,d), or mechanosensitive C-fibre neurons innervating the genitalia (Extended Data Fig. 4). We next investigated the possibility that Krause corpuscles contribute to mating behaviours of awake, naturally behaving male and female *TrkB*^*cKO*^ animals and their littermate controls, without the confounds of spinalization (Supplementary Video 3). Although the deletion of *TrkB* in *TrkB*^*cKO*^ mice is not restricted to genital afferents, these animals are overtly normal, with mutants exhibiting no deficit in gait or hairy skin mechanosensitivity^15^ (Extended Data Fig. 9a,b).

We found that *TrkB*^*cKO*^ male mice lacking Krause corpuscles were motivated to mate to a comparable extent to control littermates, indicated by a similar percentage of time that *TrkB*^*cKO*^ and control male mice spent exploring and sniffing wild-type, hormonally primed females (Fig. 5g). *TrkB*^*cKO*^ males also displayed normal mounting motion (Fig. 5h), suggesting that general motor coordination for sexual behaviour is normal in the mutants. However, *TrkB*^*cKO*^ males exhibited impaired intromission, the stage of mating when the penis is inserted into the vagina, observable as rhythmic thrusts. Compared with the controls, *TrkB*^*cKO*^ male mice displayed shorter bouts of intromission, a delayed start of intromission, a reduced total amount of intromission time and a trend towards increased interintromission intervals over successive trials (Fig. 5i and Extended Data Fig. 9c–i), suggestive of aberrant sensory feedback from the penis. Moreover, fewer *TrkB*^*cKO*^ male mice achieved ejaculation within the 75 min session compared with the control mice (Fig. 5j). Thus, Krause corpuscles of the penis are necessary for normal intromission and ejaculation.

**Extended Data Fig. 10:**
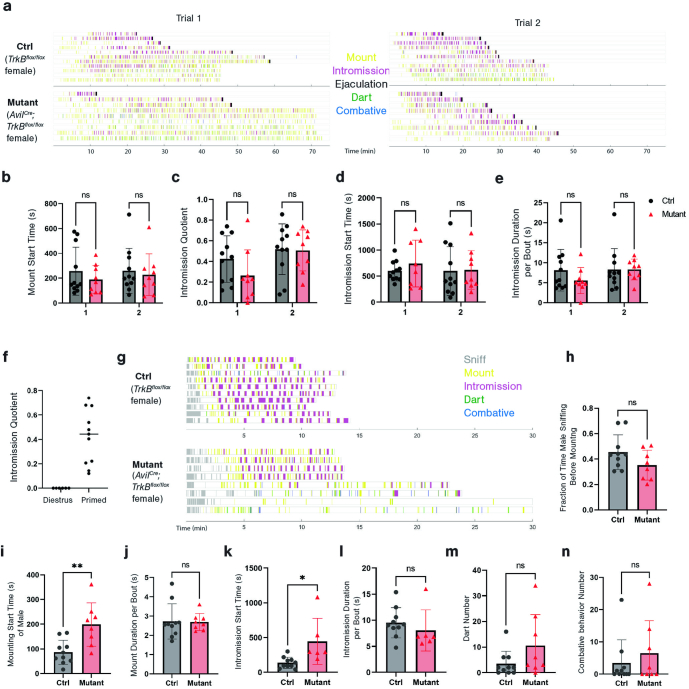
*TrkB*^*cKO*^ females display deficits in mating behaviours. **a**, Ethograms of mating behaviours of hormone-primed *TrkB*^*cKO*^ (n = 9) and littermate control (n = 11) females within a 75 min testing period across two trials, with each trial two weeks apart. **b**-**e**, Quantifications of mount start time (**b**), intromission quotient (total intromission time/(total intromission time + total mount time)) (**c**), intromission start time (**d**), and average intromission duration per bout (**e**), within each trial. Unpaired t-test, p > 0.05. **f**, Comparison of intromission quotient of wild-type females in dioestrus (without hormone priming) and with hormone priming. **g**, Ethograms of mating behaviour of naturally-cycled *TrkB*^*cKO*^ (n = 8) and littermate control (n = 10) females. Females were separated from males 10 min after the start of intromission to avoid pregnancy. If no intromission occurred, the session was stopped at 30 min. **h**-**n**, Quantifications of the fraction of time that a male mouse spends sniffing the female mouse before mounting (**h**), mounting start time (**i**), mount duration for each bout (**j**), intromission start time (**k**), intromission duration for each bout (**l**), and the number of darting (**m**) and combative behaviours (**n**) during mating for naturally-cycled *TrkB*^*cKO*^ (n = 8) and littermate control (n = 10) females. Unpaired t-test, * p < 0.05, ** p < 0.01. Error bars, s.d. Source Data

We also assessed the mating behaviours of *TrkB*^*cKO*^ female mice, which lack Krause corpuscles of the clitoris. Notably, while no differences were observed in the performance of hormonally primed, inexperienced mutant females across any behavioural measures (Extended Data Fig. 10a–f), mating behaviours of experienced females while naturally cycling during proestrus or oestrus were markedly aberrant. We observed a longer latency for receptivity to intromission, a reduced proportion of male mounting attempts that result in successful intromission (intromission quotient), and less total intromission time between *TrkB*^*cKO*^ female and wild-type male mice, compared with control female and wild-type male mice (Fig. 5k–m and Extended Data Fig. 10g–n). Thus, the loss of the dense population of Krause corpuscles in the clitoris leads to reduced sexual receptivity of experienced female mice.

## Discussion

Our findings show that Krause corpuscle afferents of the mouse genitalia are low-threshold, rapidly adapting mechanoreceptors. These neurons are optimally sensitive to 40–80 Hz mechanical vibrations, which are comparable to vibration frequencies of devices used for human sexual stimulation^43^. Similar vibration frequencies were also prominent in our measurements of tissue microvibrations generated during simulated genital skin contact (Extended Data Fig. 7e,f). Thus, while other DRG neuron subtypes innervate the genitalia (Extended Data Fig. 4) and may contribute to sexual behaviours^44^, Krause corpuscle afferents are exquisitely sensitive to low-force mechanical vibrations acting on the genitalia during sexual behaviour.

Notably, vibrotactile signals emanating from Krause corpuscles are conveyed to the DGC region of the L6–S2 spinal cord, which is distinct from the site of termination of afferents innervating adjacent hairy skin, supporting a unique role of the DGC in processing tactile signals emanating from the genitalia. Rostral to the DGC region, in male animals, the spinal ejaculation generator (SEG) lies in close proximity to the central canal of the L2–L4 spinal cord^45–47^. Although direct projections from Krause corpuscle afferents to the SEG were not observed (Extended Data Fig. 5d–g), it is possible that spinal neurons located within the DGC relay genital sensory signals to the SEG. Moreover, the SEG, along with projections from the DGC, may modulate preganglionic autonomic neurons and pudendal motoneurons in the lateral and ventral horn of the spinal cord that control erection and ejaculation^20,45–48^. Future work discerning the DGC neuronal types receiving synaptic inputs from Krause corpuscle RA-LTMRs may help to elucidate the spinal circuits that underlie sexual reflexes.

Whole-mount imaging of Krause corpuscles revealed a comparable number of these vibrotactile end organs in the male and female genitalia; however, the clitoris has an extremely high corpuscle density due to its much smaller size. This observation suggests the existence of a common innervation pattern of the penis and clitoris during early stages of genital development, followed by divergent genital tissue growth that leads to a highly sexually dimorphic density of Krause corpuscles in adulthood.

Finally, our functional experiments show that vibrotactile signals conveyed by Krause corpuscle afferents evoke sexual reflexes in both male and female mice. During mating behaviour of male mice, it is likely that olfactory cues that initiate mounting also evoke erection^49,50^, while vibrotactile inputs from the genitalia may engage the spinal sexual reflex circuitry to maintain erection during intromission. Consistent with this idea, although male mice lacking Krause corpuscles showed normal sniffing and mounting behaviours, deficits in intromission were observed (Fig. 5g–j). Moreover, given the prevalence of Krause corpuscles in the corpus cavernosa of the penis (Fig. 1l and Extended Data Fig. 1f), which greatly expand in size during erection (Supplementary Videos 1 and 2), the erectile state may augment genital sensation by altering the firing properties of Krause corpuscle afferents^12^. Relatedly, in female mice, activation of Krause corpuscle afferents elicits a clitorovaginal reflex, and this may augment afferent responses to mechanical stimuli during mating, consistent with our observation that Krause corpuscles are required for sexual receptivity of experienced female mice (Fig. 5k–m). Determining how signals emanating from Krause corpuscle RA-LTMRs are conveyed from the spinal cord to the brain to shape sexual behaviour is an intriguing direction stemming from this research.

## Methods

### Mouse lines

Mice were handled according to protocols approved by the Harvard Medical Area Standing Committee on Animals and are in accordance with federal guidelines. Female and male adult mice were used for the experiments. Mice were housed in a temperature-controlled and humidity-controlled facility, maintained under a 12 h–12 h light–dark cycle, and were given food and water ad libitum. All of the mice used in this study have been previously described, including *TrkB*^*creER*^ (JAX, 027214)^13^, *Ret*^*creER*^ (MGI, 4437245)^14^, *Advillin*^*cre*^ (JAX, 032536)^51^, *Advillin*^*FlpO*^ (ref. ^52^), *Ret*^*CFP*^ (MGI, 3777555)^53^, *TrkB*^*flox*^^54^, *Brn3a*^*cKOAP*^ (JAX, 010558)^55^, *Tau*^*FSF-iAP*^ (ref. ^56^), *Mrgprd*^*GFP*^ (ref. ^57^), *Th*^*2A-creER*^ (ref. ^52^), *Mrgrpb4*^*cre*^ (ref. ^58^), *PLP*^*eGFP*^ (JAX, 033357)^59^, *Piezo2*^*smFP-Flag*^ (ref. ^31^), *R26*^*FSF-LSL-ReaChR-mCitrine*^ (JAX, 024846), Ai80 (*R26*^*FSF-LSL-CatCh*^; JAX, 025109), Ai14 (*R26*^*LSL‐tdTomato*^; JAX, 007914)^60^, Ai65 (*R26*^*FSF‐LSL‐tdTomato*^; JAX, 021875)^61^, Ai96 (*R26*^*LSL-GCaMP6s*^)^61^ and Ai148 (*TIGRE*^*LSL-GCaMP6f-tTA2*^)^62^. All lines were kept on a mixed background, while *Advillin*^*cre*^ and *TrkB*^*flox*^ mice were bred from a mixed background to the C57Bl/6 background for two generations for mating behaviour testing. For *Advillin*^*cre*^;*TrkB*^*flox/flox*^ mice, non-neuronal recombination in the ear or tail was routinely detected, probably due to the leaky expression of *Advillin*^*cre*^. However, the observed non-neuronal recombination of the *TrkB*^*flox*^ allele was not a result of germline deletion of *TrkB*, which is lethal^63^.

### Tamoxifen treatments

Tamoxifen was dissolved in 100% ethanol, diluted 1:2 in sunflower seed oil and vacuum-centrifuged for 30 min. The TrkB^+^ population was densely labelled for immunohistochemistry and physiology with an intraperitoneal injection of 0.5 mg of tamoxifen at P5 and sparsely labelled for AP and immunohistochemistry with 0.002–0.005 mg of tamoxifen. In some cases, tamoxifen (3 mg) was administrated to the pregnant mother at E14.5 or E15.5 through oral gavage, to label the same populations^15^. The Ret^+^ population was densely labelled with 3 mg of tamoxifen delivered to the pregnant mother through oral gavage at E11.5 or E12.5 and sparsely with 0.5 mg at E12.5.

### Perfusion and post-fixation

Mice were anaesthetized with isoflurane and transcardially perfused with approximately 15 ml of 1× PBS with heparin (10 U ml^−1^) and fixed with approximately 15 ml of 4% paraformaldehyde (PFA) in 1× PBS. Once perfused, the vertebral column and brain, if needed, were removed and post-fixed in 4% PFA in PBS at 4 °C overnight. In male mice, genital tissue, including the external prepuce, was removed at the base of the penis after an incision in the scrotal area. In female mice, the entire perineal area was removed from above the protrusion of hairy skin (homologous to the “external prepuce” in males) to below the vaginal opening. The prostatic urethra, from the bladder to the base of the penis, in male mice, and the vaginal canal in female mice were roughly dissected. Genital tissue was post-fixed in Zamboni solution (phosphate-buffered picric acid-formaldehyde) at 4 °C overnight, and tissues were washed in 1× PBS the next day.

### Fixation of the erection state

The penis was collected after the mouse was freshly perfused with 1× PBS with heparin and the penis was isolated from the prepuces. One suture was fastened around the proximal base of the penis (proximal corpus cavernosum, not the corpus cavernosum glans) and the other was loosely wrapped around the base of the glans, just distal to the 90° bend. A 30 G needle attached to a 3 ml syringe containing Zamboni solution or 2% PFA was inserted between the sutures. Care was taken to ensure that the needle stayed inside the glans penis. Gentle pressure was applied to the syringe until the penis assumed a cup shape and, while maintaining pressure on the syringe, the distal suture was fastened. As the syringe was removed, the sutures were further tightened, and the tissue was post-fixed in Zamboni solution at 4 °C overnight. Subsequently, the tissue was transferred to 30% sucrose at 4 °C until it sank to the bottom, followed by embedding in OCT for cryosectioning.

### Immunohistochemistry

Spinal cords and DRGs were isolated with forceps after removal of the dorsal and ventral vertebral column, and nodose ganglia were dissected after removal of the overlying submandibular glands and musculature. Penile tissue was isolated from the preputial glands and external and internal prepuces, and the proximal portion of the penis was discarded. Working proximal to distal, the clitoris was isolated from perineal tissue by removal of remaining pelvic musculature and preputial glands, then by fine dissection of the clitoris and urethra from above the vaginal canal and inside of the external prepuce. Glabrous digit-tips were cut from the paws for staining.

Tissue was cryoprotected in 30% sucrose in 1× PBS at 4 °C overnight, embedded in Neg-50 and frozen at −80 °C. Spinal cord and DRG samples were sectioned transversely using a cryostat (Leica) at 30 µm and placed onto slides. Genital tissue and glabrous digit-tips were sectioned sagittally at 30 µm thickness and placed onto slides or at 200 µm and placed into a well plate with 1× PBS. Thin cryosections were allowed to dry overnight at room temperature. The sections were rehydrated with 1× PBS, blocked with 5% normal donkey serum in 0.1% PBST (0.1% Triton X-100 in 1× PBS) for 2 h at room temperature, and incubated with primary antibodies in blocking solution for 2 days at 4 °C. The slides were rinsed in PBST three times for 10 min and incubated with secondary antibodies in blocking solution overnight at 4 °C. The slides were then rinsed in PBST 3 times for 10 min and mounted with Fluoromount-G. The sections were imaged on the Zeiss LSM 700 or 900 confocal microscope.

Immunohistochemistry of thick sections was performed using previously described protocols for whole-mount tissue^56^. Sections were washed in 0.3% PBST five times for 1 h and incubated in primary antibody in blocking solution (5% normal donkey serum, 75% PBST, 20% DMSO) at room temperature for 3–5 days under gentle agitation. After five 1 h washes with 0.3% PBST, secondary antibodies in blocking solution were added for 2–4 days at room temperature. The sections were then washed twice in 0.3% PBST for 1 h, stained with DAPI (1 µg ml^−1^, 5 min) to visualize nuclei, further washed three times in 0.3% PBST for 1 h, and dehydrated in serial methanol concentrations (50%, 75%, 100%, 1 h each) and then overnight in 100% methanol. Tissue was cleared in BABB (1:2, benzyl alcohol: benzyl benzoate) and imaged on the Zeiss LSM 700 or 900 confocal microscope or AxioZoom stereoscope while fully submerged. Dehydrated tissue was stored in methanol at 4 °C.

The primary antibodies used in this study were as follows: chicken anti-GFP (Aves Labs, GFP-1020, 1:500), goat anti-GFP (US Biological, G8965-01E, 1:500), goat anti-mCherry (CedarLane, AB0040-200, 1:500), rabbit anti-CGRP (Immunostar, 24112, 1:500), chicken anti-NF200 (Aves Labs, NFH, 1:200), rabbit anti-NF200 (Sigma-Aldrich, N4142-.2ML, 1:500), rabbit anti-S100 (ProteinTech, 15146-1-AP, 1:200–1:500), rat anti-TROMA-1 (DSHB, AB_531826, 1:200), goat anti-CD31 (R&D Systems, AF3628, 1:500), guinea pig anti-Flag (1:500)^31^ and IB4 (Alexa 647 conjugated) (Invitrogen, I32450, 1:500).

### Quantification of corpuscle structure and distribution

To determine the total number of corpuscles per animal, care was taken in collecting all of the 200 µm sections from a given clitoris or penis sample and staining them with antibodies for S100 and NF200. All of the sections were imaged under a confocal microscope. The number of corpuscles in each section was manually counted using ImageJ, with the location of each corpuscle preserved in an ROI file. The borders of imaged tissue were manually outlined and saved. Each section’s volume was computed by multiplying the area of the outlined region by the height of the *z* stack, allowing for calculation of corpuscle density. To generate distribution heat maps, the ROI files containing the location of each corpuscle and the tissue outline were imported into MATLAB. The distribution of corpuscles was binned in a grid, and a Gaussian filter was used to smooth the density distribution.

Complex Krause corpuscles were defined as Krause corpuscles containing tightly coiled axons. Complex Krause corpuscles often exhibited globular shapes, while some also exhibited elongated shapes with convoluted axonal profiles. Simple Krause corpuscles were defined as the corpuscles containing 1 or 2 linear (non-coiled) axons. All simple corpuscles have an elongated shape, thereby exhibiting some similarity with Pacinian corpuscles but with a much smaller size.

### Transmission electron microscopy

Adult mice were intracardially perfused with phosphate buffer and a glutaraldehyde/formaldehyde fixative. The thickest segment of the clitoris was isolated and the distal third of the glans penis was microdissected from the internal tissue and flattened on a piece of filter paper for overnight post-fixation at 4 °C. After the samples were washed in 0.1 M phosphate buffer (pH 7.4), they were cryoprotected overnight in 30% sucrose in 0.1 M phosphate buffer. After samples were embedded in Neg-50 Frozen Section Medium, they were frozen and cryosectioned into 100 µm sections and placed into the 0.1 M phosphate buffer solution. Medial sections of the clitoris and complete sections of the glans penis were selected for sample preparation.

The samples were osmicated in cacodylate buffer with 1% osmium tetroxide/1.5% potassium ferrocyanide for 1 h. The sections were washed with double-distilled H_2_O and stained in a solution of 0.05 M sodium maleate (pH 5.15) and 1% uranyl acetate at 4 °C overnight. After washing with double-distilled H_2_O, the sections were dehydrated with serial ethanol concentrations and propylene oxide. The sections were then infiltrated with 1:1 mix of epoxy resin (LX-112, Ladd Research) and propylene oxide at 4 °C overnight. The sections were embedded in an epoxy resin mix and cured at 60 °C for 48–72 h. Ultrathin (approximately 60 nm) sections were generated and imaged on the JEOL 1200EX transmission electron microscope at 80 kV accelerating voltage. The images were cropped using ImageJ.

### Whole-mount AP staining of skin and spinal cord

TrkB^+^ and Ret^+^ afferents were sparsely labelled using the *Brn3a*^*cKOAP*^ placental AP (PLAP) reporter mouse^55^, as described above, to enable visualization of single nerve terminals in genital and glabrous tissue. C-LTMRs were densely labelled using *Th*^*2A-creER*^;*Brn3a*^*cKOAP*^ animals, with tamoxifen (2 mg intraperitoneal (i.p.)) administered at 3 weeks old. Although sympathetic fibres are TH^+^, they do not express BRN3A; thus, sympathetic fibres are not labelled in *TH*^*2A-creER*^*;Brn3a*^*cKOAP*^ animals. The MRGPRB4^+^ afferents were labelled using intrathecal injections of AAV-FLEX-PLAP^64^ (5 μl, titre 1.2 × 10^13^ genome copies per ml) into 4-week-old *Mrgprb4*^*cre*^ mice^58^. All of the mice were perfused after 7 weeks of age.

Different dissection methods were used for different types of tissue: (1) for female genital tissue, after the clitoris dissection described above, a cut was made along the ventral midline of the urethra to allow spreading of the wings of the distal clitoris. (2) For male genital tissue, to permit comprehensive visualization of penile innervation, the superficial layer together with the corpus cavernosum of the glans penis was carefully removed from the internal mesenchyme including the os penis, and separated into two pieces. While not included in our primary analysis, the internal prepuce of the penis was retained for whole-mount AP staining. To isolate the prostatic urethra, also excluded from our primary analysis, the seminal vesicles and lobes of the prostate were removed, and a cut was made from the distal urethral opening to the opening of the bladder to permit flattening of the tissue. (3) For glabrous skin, the entire ventral surface of the paw was dissected from the underlying tissue for staining. (4) For hairy skin, the hairs on the back and around the genitalia were removed using Nair. (5) For spinal cord and dorsal column nucleus, the whole spinal cord was dissected with the dorsal column nucleus attached, and the overlying dura was removed.

The post-fixed and dissected tissue was incubated in PBS at 68 °C for 2 h to inactivate endogenous AP, then rinsed three times for 5 min in B3 buffer (0.1 M Tris pH 9.5, 0.1 M NaCl, 50 mM MgCl_2_, 0.1% Tween-20) at room temperature. For the PLAP enzymatic reaction, tissue samples were incubated at room temperature overnight in BCIP and NBT in B3 buffer (3.4 µl of each per 1 ml of B3 buffer). Stained tissue was then pinned flat in a dish, fixed in 4% PFA in PBS for 1 h at room temperature and serially dehydrated in ethanol (50%, 75%, 100%, 1 h each, then 100% overnight) while covered. Tissue was then cleared and imaged in BABB using the Zeiss AxioZoom stereoscope.

### Quantification and reconstruction of single-neuron morphology

Arborizations belonging to individual neurons were imaged for analysis. The terminal area was measured in ImageJ by drawing a tight polygon around the terminals of a given axon, and the number of corpuscles innervated by each fibre was counted manually. It is possible that the corpuscles of a given afferent occupy a larger volume in the *z* dimension than is measured in this manner. Simple corpuscles were deemed to be thin and linear terminals, while complex corpuscles were identified as having wider and bulbous terminals. Reconstruction and filling of representative fibres was performed using the ImageJ SNT plugin.

### Genital skin injections

Young adult mice were anaesthetized with continuous inhalation of 2% isoflurane from a precision vapourizer for the duration of the procedure (5–10 min). Injections were done with a bevelled borosilicate or quartz glass pipette. The glass pipette was connected to an aspirator tube assembly (Sigma-Aldrich, A5177-5EA), which was connected to a syringe to control the air pressure.

For penis injections, pressure was applied near the genital region to fully externalize the glans penis from the external prepuce and internal prepuce. A pair of blunt forceps was used to stabilize the penis, while inserting the glass pipette into the distal end of penis. The penetration depth ranges from superficial to 3 mm deep to label the whole glans penis. Pressure was applied to the syringe to eject the liquid inside the glass pipette. After injection, the pipette was kept inside the tissue for 10 s to reduce the leaking. A total of 3–4 locations on the penis were injected. For successful injections, the fast green dye mixed in the liquid was visible inside the tissue for at least 10 min. Rapid dissipation of the fast green dye (within seconds) indicated a failed injection.

For clitoris injections, hairs near the genital protrusion (hairy skin) were removed by Nair treatment and subsequent cleaning using 70% ethanol. A pair of blunt forceps was used to stabilize the protrusion while inserting the glass pipette 1–2 mm deep into the middle part of protrusion. Care was taken to ensure that the needle did not impale the urethra. After injection, the pipette was kept inside the tissue for 30 s to minimize leak into the hairy skin region. An injection into the clitoris was considered to be successful if the fast green dye mixed in the liquid was visible below but not on the surface of hairy skin.

For injection of mid-line hairy skin, hairs near the genital protrusion in either male or female were removed by Nair treatment and subsequently cleaning using 70% ethanol. A pair of blunt forceps was used to stabilize the hairy skin while inserting the glass pipette into the superficial hairy skin. After a successful injection, the fast green dye was immediately visible within the hairy skin region during the injection.

A total volume of 2 μl was injected into either the male or female target region, for injection of either CTB or AAV. For CTB injections, 4–8 week old animals were used, and the injected animals were perfused in 3–4 days. For AAV injections, 3–4 week old animals were used, AAV2-retro-hSyn-FlpO (4.95 × 10^13^, Boston Children’s Hospital Viral Core) was used, and the injected animals were perfused after 3 weeks.

### Spinal-cord terminal quantification

Owing to the characteristic decrease in the size of the dorsal column from the lumbar enlargement to more caudal regions of the spinal cord, the axial levels of the lumbosacral spinal cord sections were determined by the ratio of the depth of dorsal column (*L*_d_) to that of the central canal (*L*_c_). *L*_d_ was calculated as the length from the midpoint of dorsal surface of spinal cord to the ventral border of dorsal column, whereas *L*_c_ was calculated as the distance between the midpoint of dorsal surface of spinal cord and the central canal. Ratios of *L*_d_/*L*_c_ from the images in the reference atlas of the mouse spinal cord (https://mousespinal.brain-map.org/imageseries/showref.html) were calculated and used as the reference to identify axial levels of the lumbosacral sections.

To average the fluorescence intensity of spinal-cord sections of the same axial level, images were rotated to align the dorsal surface of spinal cord horizontally, and the midlines of the spinal cords were aligned using MATLAB. A region of interest (750 μm^2^) on the dorsal and medial regions of spinal cord sections was selected from each image for averaging. The fluorescence intensity in the square region of interest was measured, downsampled to 100 × 100 px and then averaged. As no fibres were seen in the dorsal column, its fluorescence intensity was used as the baseline and subtracted from the average intensity.

### In vivo MEA recordings of L6 DRG neurons

Adult male mice (>6 weeks) were anaesthetized with inhaled isoflurane (approximately 2.0%) through a nose cone for the duration of the experiment. Body temperature was maintained at 37 ± 0.5 °C using a custom-made surgical platform equipped with two heating pads mounted on acrylic. An adjustable gap (~1 cm wide and ~7 cm long) between the heating pads allowed for access to genital stimulation from below.

After induction of anaesthesia, pressure was applied to the abdomen and perineal skin to fully externalize the glans penis. To maintain the externalized state, a minimal amount of Vetbond tissue adhesive was applied to the retracted internal prepuce and the proximal base of glans penis to prevent retraction. Subsequently, the back of the mouse was shaved, and a midline incision was made over the lumbar and sacral vertebrae. A custom-made spinal clamp was applied to the L5 vertebra to secure the spinal column. The paravertebral muscles over the L6 spine were dissected, and the bone covering bilateral L6 DRG was removed with a bone drill. Surgifoam sponges and cotton were used to control bleeding. After cleaning the surface of the L6 DRG, the epineurium surrounding the DRG was removed with fine forceps, then saline was added on the top of the DRG.

The platform was then moved to the MEA recording setup. A 32-channel silicon probe (Cambridge NeuroTech, ASSY-37 H6b) was inserted into either the left or right L6 DRG. The signals were amplified and recorded using an Intan Technologies RHD2132 amplifier chip and RHD USB Interface Board. Data acquisition was controlled with open-source software (Intan Technologies Recording Controller v.2.07).

After insertion of the MEA probe into the DRG, brush stimuli using a cotton swab or paintbrush were applied to the glans penis and perineal hairy skin to search for penis-innervating neurons. Neurons that exhibited robust firing in response to mechanical stimulation of the penis but not the adjacent hairy skin were classified as penis-innervating neurons.

For the animals with opsin expression in sensory neurons, the receptive field on the penis was identified, then a fibre optic probe was oriented to deliver light pulses to the penis to optotag sensory neurons expressing opsin. The light pulse was generated by a fibre-coupled LED (M470F4, Thorlabs) with an LED driver (LEDD1B, Thorlabs), and light pulses had a duration of 1 ms and a frequency of 10–20 Hz. Once entrainment of spiking to light pulses was confirmed, indentation was delivered using a mechanical stimulator (300C-I, Aurora Scientific) with a custom-made indenter using a probe with a ~200 µm tip diameter. Owing to the physical constraint of the set-up, only the dorsal side of the penis was accessible for indentation. If the receptive field was on the dorsal side of the penis, the indenter tip was adjusted to the centre of receptive field, vertical to the surface. A curved piece of plastic was positioned on the ventral side of the penis to support the indentation. A series of step indentations ranging from 1 mN to 75 mN was applied, with each step lasting for 0.5 s. A minimum of 20 repeated trials was conducted. Next, a series of sine-wave vibrations ramping from 0 to 20 mN at varying frequencies (from 10 Hz to 120 Hz) were applied using the indenter. The vibration stimuli of different frequencies were randomized in order, and each frequency was repeated five times. The force and displacement of the indenter were commanded with custom MATLAB (v.2019a or v.2021a) scripts controlling a data-acquisition board (National Instruments, NI USB-6343).

JRCLUST was used to automatically sort action potentials into clusters, which were then manually refined and classified as single or multi units (https://github.com/JaneliaSciComp/JRCLUST).

To calculate the conduction velocity of optotagged neurons, the latency was determined by subtracting the time of each spike from the middle point of each light pulse. The latencies for the four optotagged TrkB^+^ Krause afferents were 4.4 ms, 4.8 ms, 7.8 ms and 13.6 ms, respectively. The length of axonal projections from L6 DRG to the genitalia in adult mice was estimated to be 5 cm. Thus, the conduction velocities of the four optotagged TrkB^+^ neurons are 11.4, 10.4, 6.4 and 3.7 m s^−1^ respectively.

To determine the mechanical thresholds for vibration stimuli across different vibration frequencies, the time of the first spike in response to the vibration in each trial was identified. The corresponding recorded force at that timepoint was defined as the mechanical threshold for that trial. Thresholds of the trials at each vibration frequency were then averaged.

### In vivo extracellular recording of L6 DRG neurons using glass electrode

The surgical procedures were similar to those in the previous section. The method for extracellular recording with a glass electrode was adapted from a recent study^65^. After exposure of the L6 DRG in anaesthetized animals, a borosilicate glass electrode filled with saline (2–3 MΩ) was slowly inserted into the DRG using a manipulator (MP-225, Sutter Instrument) while searching for neurons responding to electrical or mechanical stimuli applied to the externalized glans penis. The data were amplified and digitized using the Multiclamp 700B and Digidata 1550B (Molecular Devices) system in the pipette potential mode with a 1,000× gain. Signals were collected at a 50 kHz sampling rate, under a 100 Hz high-pass filter and a 3 kHz Bessel filter.

For electrical stimuli, a custom-made bipolar electrode was placed on the top of the dorsal nerve of the penis, delivering 1 mA pulses of 0.2 ms duration, to activate most or all sensory fibres innervating the glans penis. When a DRG unit showed reliable responses to the electrical stimulation, the glass recording electrode was repositioned to maximize the size of extracellular spike. The mechanical receptive field of the unit was then explored using a fine paint brush and a custom-made mechanical stimulator that was previously described^65^. Subsequently, a series of step indentations and, in some experiments, vibrations were applied to the centre of receptive field to assess the mechanical threshold, adaptation properties or vibration tuning properties. The stimulation was programmed and the recordings were triggered using MATLAB.

The latency to spike was determined by the interval between the start of the electrical stimulation artifact and the onset of the spike signal. The threshold of each unit was defined as the minimum force needed to evoke a response from the neuron. Units that responded to electrical stimuli of the glans penis but not to step indentation stimuli were classified as non-responsive.

### In vivo calcium imaging of L6 DRG neurons

The surgery procedure for male mice was the same as for the MEA recordings above. After the L6 DRG was exposed, the platform was moved to an upright epifluorescence microscope (Zeiss Axio Examiner) with a ×10 air objective (Zeiss Epiplan, NA = 0.20) for imaging. The light source was a 470 nm LED (M470L5, Thorlabs) with an LED driver (LEDD1B, Thorlabs), and a CMOS camera (CS505MU1, Thorlabs) was triggered at 10 frames per second with 50 ms exposure time using ThorCam software (v.3.7.0). All stimuli were synchronized with the camera using a data-acquisition board (National Instrument, NI USB-6343).

Indentation on the penis was performed in a similar method as described above for the MEA recordings, with a few modifications. The indentation step was increased to 3 s to allow for the assessment of adaption properties. The interval between each step was increased to at least 8 s to accommodate for the slow-decay dynamics of the GCaMP signals. The duration of ramping sinewave vibration was also increased to identify the mechanical thresholds using different vibration frequencies.

For thermal stimulation of the penis, a water reservoir device, inspired by a previously described method^66^, was used. This device consisted of a reservoir connected to water baths at different temperatures, including room temperature, ice water (~4 °C) and hot water (~55 °C). The penis was submerged in the water reservoir. Then, water of different temperatures was pumped into the reservoir at a controlled speed, and room-temperature water was used during the baseline measurements. The water overflowing from the reservoir was directed to a collection chamber located beneath the reservoir. The temperature in the reservoir was monitored using a thermocouple microprobe (IT-1E, Physitemp) and a thermometer (BAT-12, Physitemp).

For clitoris stimulation, the indenter tip was placed vertically on the surface of protrusion where the clitoris was located. To minimize the dorsal–ventral movement of mouse’s lower body during indentation, the mouse tail was held down using a clamp. Similar step indentation and vibration stimuli were used for clitoris as described for the penis. After the mechanical stimulation, the surgery chamber was removed from the microscope, and the anaesthetized mouse was placed in a supine position. An incision was created on the protrusion to expose the dorsal nerve of clitoris, and saline was added on the top of the nerve. Subsequently, the mouse was reoriented to the prone position, with the spinal clamp reattached. After locating the same field of view of the DRG, a pair of custom-made bipolar electrodes (silver wire, with ~1 mm distance between the two points) was placed on the dorsal nerve of clitoris, and a train of 1 ms pulses (10 Hz, less than 1 mA) was then delivered.

For the calcium imaging analysis, motion correction and spatial high-pass filtering was conducted using a custom-written ImageJ macro code that used the ImageJ plugin moco and the Unsharp mask filter, and then regions of interest were manually selected. Cells exhibiting baseline signal and/or calcium responses were identified and aligned across videos encompassing various stimuli. The intensity measurements generated by ImageJ were then processed for further analysis using MATLAB. For the calculation of Δ*F*/F, F was determined using the baseline activity (average fluorescence intensity before each stimulation). The mechanical threshold for each step indentation session was determined on the basis of the first discernible calcium spikes aligned with the step indentation. To determine the mechanical thresholds for vibrations at varying frequencies, the s.d. of the baseline activity was calculated and the threshold for the response was defined as 5 × s.d. above the baseline. Next, the recorded force at that timepoint was defined and averaged across trials with the same frequency. To differentiate between clitoris-innervating neurons and neurons innervating hairy skin, only neurons that responded to electrical stimulation (5 × s.d. above the baseline) were included for further analysis.

### Sticky-tape assay

The sticky-tape assay as a measure of hairy skin sensitivity was performed using a previously described protocol^67^. A piece of common laboratory tape (2 cm by 2 cm, Fisher Scientific, 15-901-5R) was secured to the back of the animal. Then, the animal was introduced into a 15 cm by 15 cm Plexiglass enclosure and video-monitored for a period of 5 min. The animal’s attempts to remove the tape, characterized by scratching, biting and body shaking, were quantified using BORIS software.

### Tactile PPI analysis

The tactile prepulse inhibition (PPI) experiments were conducted according to a previously described procedure^68^. The mouse was positioned inside a plastic cylinder with 3.8 cm diameter. The container was then secured in a soundproof chamber (SR-LAB Startle Response System). Before an acoustic startle stimulus (125 dB, 20 ms), a 0.9 psi air puff (50 ms) as a prepulse was delivered to the back of the animal at various interstimulus intervals (50, 100, 250, 500 and 1,000 ms). The startle response of the animal was recorded using an accelerometer attached to the chamber. PPI was calculated as %PPI = [1 − (startle response from pulse following prepulse/startle response from pulse alone)] × 100.

### Power spectrum analysis of genital contact

Force was measured using a load cell (Model MBL, 50 g, Honeywell) connected to a strain gauge amplifier (DMD-465WB, Omega). Vibratory stimuli were administered directly onto the load cell. For the force measurements during genital contact, the glans penis, dissected from a perfused mouse, was moved across a piece of freshly dissected vaginal tissue affixed to the load cell at a speed of around 1–2 cm s^−1^, simulating genital contact during mating. The data were digitized at a sampling rate of 50 kHz using the Digidata 1550B (Molecular Devices) system, after noise reduction using the Hum Bug Noise Eliminator (Digitimer). Power spectrum analyses were conducted with Clampfit software (v.11.2), with fixed-duration periods (0.3 s) sampled from either the baseline or during stimulation phases.

### Spinal transection

Mice were anaesthetized with inhaled isoflurane (approximately 2.0%) through a nose cone throughout the surgery. The bladder was emptied by applying gentle abdominal pressure. Body temperature was maintained at 37 ± 0.5 °C using a heating pad, and ophthalmic ointment was placed on the eyes. Once reflexes were absent, the back was shaved and sterilized with alternating swabs of ethanol and betadine. A midline incision was made over the thoracic spinal column, and the paravertebral musculature was cut to expose the gap between the T9 and T10 spinal segments. A curved scalpel blade (10012-00, FST) was inserted between the T9 and T10 spinal segments and moved laterally to ensure thorough transection. Bleeding was controlled with Surgifoam sponges and cotton. The skin of the back was sutured and mice were allowed to recover on a heating pad. Hindlimb paralysis was assessed to confirm success of the spinal transection. Food and DietGel were placed onto the floor of the cage, and carprofen (5 mg per kg) was injected subcutaneously every 24 h. Bladder expression was performed every 2–3 h on the day of surgery and every 6–8 h on the next day. The mice were euthanized within 3 days of spinal transection.

### Male sexual reflex behaviours

Reflex behaviours were tested at 6 or 24 h after spinal transection. The awake animal was restrained in a round acrylic chamber in a supine position, with the paralysed hindlimbs secured on the platform using Scotch tape. Two cameras were positioned from different angles, focusing on the genital region of the mouse. To begin the procedure, pressure was applied to the side of the genital region to fully externalize the glans penis of the mouse. Reflexes might have occurred during or right after the externalization due to the force applied in this process, but this effect would end within 1 min of externalization.

For evoking vibration-induced sexual reflexes in male mice, 2 min after externalization of the glans penis, vibration stimuli (50 Hz) were delivered using a custom-made mechanical stimulator as previously described^65^. The stimulator consisted of a DC motor connected to an arm (10 mm long) with a round tip (2 mm in diameter) mounted on the end. The motor was driven by a custom-built current supply controlled by a data-acquisition board (NI USB-6343, National Instrument). The force was calibrated using a custom-made force sensor. The stimulator was attached to an articulating arm that could be moved manually. Before applying vibration stimuli, the stimulator tip was positioned close to the lateral side of glans penis, while a holder attached to an articulating arm was moved to the opposite side to ensure the glans penis remains in place. The vibration stimuli were delivered as a 50 Hz sine wave, with a 1 s duration followed by a 1 s interstimulus interval. Each session consisted of 10 such epochs, lasting a total of 20 s. The stimuli were programmed and delivered using MATLAB. Vibration sessions were spaced 2–3 min apart, and three vibration sessions were conducted for each animal.

For brush-induced sexual reflexes, 2 min after externalization of the glans penis, a cotton swab was used to apply brush stimuli in either the proximal or distal direction. Each session lasted 20 s, with intervals of longer than 2 min between sessions.

Optogenetic induction of sexual reflexes was performed 2 min after externalization of the glans penis of spinalized animals. The optic fibre (diameter, 1,000 µm; 0.39 NA; M35L02, Thorlabs) connected to a fibre-coupled LED (M470F4, Thorlabs) was directed towards the glans penis. The stimulation sessions lasted 20 s, with 10 Hz and 2 ms pulses used for animals expressing ReaChR, and 20 Hz and 1 ms pulses used for animals expressing CatCh. The maximum light intensity was 10 mW. Mice that showed excessive spontaneous activity before optogenetic stimulation, probably due to a blockage in the bladder, were excluded.

For mechanically induced sexual reflex behaviours, 2–4-month-old mice were used. For the optogenetic stimulation of *TrkB*^*creER*^ mice, 4–6-month-old animals were used because externalization of the glans penis was not feasible in younger animals that received tamoxifen treatments at P5. Red light was used to illuminate the set-up during optogenetic induced behaviours, while room light was used for mechanical stimulation measurements. Videos were taken at 60 fps using SpinView (v.1.1.0.43). To quantify the sexual reflexes, the start and end of each reflex behaviour was manually labelled using BORIS software^69^. The behavioural measurements were then exported, analysed and plotted in MATLAB.

### Female sexual reflex behaviours

Two days before testing, female mice were treated with 17β-estradiol benzoate (35 μg delivered i.p.) to facilitate access to the vaginal opening. The day before testing, mice were spinal transected at the T9/T10 level, using methods described above. On the day of the test, the awake, paralysed animals were gently restrained in a supine position with all four limbs taped on a platform, to reduce motion artifacts during the pressure measurements. A latex balloon (Harvard Apparatus, 73-3478) affixed to a cannula (PE-50 tubing) was carefully inserted into the vagina, and secured with the cannula taped on the platform. The assembly was connected to a pressure transducer (BIOPAC system, TSD104A) through saline-filled tubing. The transducer’s output was subsequently amplified and digitized at a 2000 Hz sampling rate using a BIOPAC MP150 system and AcqKnowledge software.

After the animal and the balloon were secured, the custom-made mechanical stimulator described above^65^ was positioned at the top of the clitoris within the genital protrusion. When a stable baseline was established, vibration stimuli were delivered as a 50 Hz sine wave, using a 1 s duration followed by a 0.5 s interstimulus interval. Each session consisted of 10 such epochs, lasting a total of 15 s. The stimuli were programmed and delivered using MATLAB. Vibration sessions were spaced more than 2 min apart, and more than three vibration sessions were conducted for each animal. Each mouse was then anaesthetized with 1.5% isoflurane, anaesthesia was verified by toe pinch and additional vibration sessions were conducted. The start of the vibration session was manually annotated in the AcqKnowledge software. As whole-body movements of the mouse were also detected during vaginal pressure measurements, each mouse was monitored throughout the experiment and all movement artifacts were marked accordingly in the software.

To measure the vaginal pressure during optogenetic activation, the hair on top of the genital protrusion was removed using Nair. The optic fibre (diameter, 1,000 µm; 0.39 NA; M35L02, Thorlabs) connected to a fibre-coupled LED (M470F4, Thorlabs) was directed towards the clitoris within the genital protrusion. The stimulation sessions lasted 20 s, with 1 ms pulses at a frequency of 20 Hz and a peak light intensity of 10 mW, while measuring vaginal pressure.

The pressure measurements were processed in MATLAB after conversion using acq-tools (https://github.com/bccummings/acq-tools). Owing to the variability in absolute pressure values across animals, as a result of the variations in the baseline pressure and positioning of the balloon, the fold increase relative to baseline noise level was used to standardize the response across animals. The baseline noise level was calculated as the s.d. of the pressure measurement during the 10 s period preceding each stimulation session. Motion artifacts caused by whole-body movements, annotated during experiments and identified by clustered sharp peaks, were distinct from the gradual changes indicative of genuine vaginal pressure. Motion artifacts were pronounced in C57BL/6 mice but were less commonly encountered with CD1 mice; we therefore used 2–4-month-old CD1 or mixed-background animals for all female sexual reflex behavioural experiments.

### Mating behaviours

At least 2 weeks before testing, mice were transferred to a reverse 12 h–12 h light–dark cycle room. All of the mice used for mating behaviours were 2–4 months old. They were from a mixed background but backcrossed to C57Bl/6 mice for two generations.

The mating behavioural analysis was similar to that of a previous study^70^. The male mouse was singly housed for at least 2 weeks before testing and was socialized with a female mouse overnight at least 1 week before the test. Then, 2 days before the test, female mice were treated with 18–35 μg of 17β-estradiol benzoate (dissolved in sterile sesame oil, final volume of 50–100 μl, subcutaneous injection) and treated again with 50–100 μg progesterone (dissolved in sterile sesame oil, final volume of 50–100 μl) 5 h before the test. During the mating test, a female mouse was placed into the home cage of the male mouse, and the cage cover was replaced by a custom-made wall (6 inches tall) to prevent mice from leaving their cage. Mating behaviours were visualized in the male’s home cage using USB cameras, which were placed above the cage capturing videos at 30 fps using IC capture (v.2.5). The behaviour room was dark, illuminated only with infrared lamps.

When testing the mating performance of the males, a wild-type female mouse that was unreceptive 10 min after the start of the session would be replaced with another female, so that only receptive females were used for testing males. Mating behaviour was monitored over video for 75 min. The male mice underwent testing for at least three rounds, with each round occurring at least 1 week apart.

When testing the mating performance of female mice, only experienced males were used. The same group of female controls and mutants were hormone-primed (as described above) in the first two rounds of the mating test, while, in the third round, they were in a natural oestrus cycle. The first two rounds were conducted 2 weeks apart, and the third trial was conducted 4 weeks after the second trial to allow for the recovery of oestrus cycle. The oestrus cycle was monitored using the vaginal cytology method according to previously established protocols^71,72^. Vaginal smears were collected, stained with crystal violet and examined to determine the oestrus stage. Only female mice in proestrus or oestrus were used for mating behaviours within 3 h of vaginal lavage. As hormone priming prevents the treated female mice from becoming pregnant, we allowed the female mice to engage in the full mating test (75 min) in the first two rounds to provide sexual experience to the cohort. In the third test for the same groups of animals but under their natural oestrus cycle, females were separated from the males 10 min after the start of intromission to avoid pregnancy. If no intromission occurred, the mating session was terminated at 30 min.

The videos of mating behaviours were manually scored using BORIS software^69^ by an experimenter who was blinded to the genotype of the animals. For male mating behavioural analysis, the following behaviours were scored: sniffing, mounting, intromission and ejaculation. Mounting refers to the males’ rapid pursuit of females, followed by grasping their rear, and often followed by short probing without gaining access to the female genitalia. Intromission represents the long rhythmic thrusting, indicating the male’s successful penetration of the vagina. The mounting and intromission periods do not overlap with the scoring methods used; mounting is considered to be the pursuit and adjustment period before the intromission period. The intromission bouts lasting less than 2 s were excluded from the analysis due to the lack of rhythmicity of movements and the possibility that penetration did not fully occur. Moreover, combative behaviours and darting behaviours of female mice were scored during the female mating behaviour assessment trials. Combative behaviour refers to the instances in which the female confronts or fights with the male, while the dart behaviour occurs when the female mouse attempts to quickly escape from the mounting of the male mice.

### Statistics and reproducibility

Data were analysed using MATLAB (v.2019a or v.2021a), Python (v.3.7.7) and GraphPad Prism 10. The number of samples and the statistical tests used for individual experiments are included in the figure legends when length permitted. In Fig. 2c, *n* = 10 sections from 3 control females, 10 sections from 3 *TrkB*^*cKO*^ females, 7 sections from 2 control males and 20 sections from four *TrkB*^*cKO*^ males. In Fig. 2f, *n* = 32 TrkB^+^ axons from 5 males, 16 Ret^+^ axons from 3 males, 26 TrkB^+^ axons from 4 females, 11 Ret^+^ axons from 3 females, 26 TrkB^+^ axons from 4 animals’ digits, and 17 Ret^+^ axons from 4 animals’ digits.

Statistical analysis was performed using unpaired *t*-tests, one-way ANOVA with post hoc multiple unpaired *t*-tests, two-way ANOVA with Tukey’s multiple-comparisons test and Fisher’s exact tests. All *P* values for unpaired *t*-test used are two tailed. The exact *P* values and other statistics for unpaired *t*-tests, multiple unpaired *t*-tests or Tukey’s multiple-comparison test used in the study are as follows: in Fig. 1g, *P* = 0.9355, *t* = 0.085, d.f. = 5, *F* = 1.002; Fig. 1i, between clitoris and glans penis, *P* < 0.000001, *t* = 13.66, d.f. = 917, *F* = 2.514; between clitoris and digit (Meissner), *P* < 0.000001, *t* = 6.82, d.f. = 682, *F* = 8.169; Fig. 2f, for datapoints of penis, *P* = 0.001138, *t* = 3.482, d.f. = 44; for datapoints of digit, *P* = 0.000224, *t* = 4.068, d.f. = 39; for datapoints of clitoris, *P* = 0.000104, *t* = 4.409, d.f. = 33; Fig. 2g, for datapoints of penis, *P* = 0.002853, *r* = 3.156, d.f. = 45; for datapoints of digit, *P* = 0.8476, *t* = 0.1934, d.f. = 41; for datapoints of clitoris, *P* = 0.00666, *t* = 2.885, d.f. = 35; Fig. 4i, between clitoris and penis in the TrkB^+^ neurons, *P* = 0.000001; between clitoris and penis in the Ret^+^ neurons, *P* = 0.2023; between TrkB^+^ and Ret^+^ neurons of clitoris, *P* < 0.000001; between TrkB^+^ and Ret^+^ neurons of penis, *P* = 0.000632; Fig. 5g, for session 1, *P* = 0.6457, *t* = 0.08194, d.f. = 15; for session 2, *P* = 0.6358, *t* = 0.4834, d.f. = 15; for session 3, *P* = 0.6994, *t* = 0.8235, d.f. = 15; Fig. 5h, for session 1, *P* = 0.8526, *t* = 0.1891, d.f. = 15; for session 2, *P* = 0.3264, *t* = 1.015, d.f. = 15; for session 3, *P* = 0.5408, *t* = 0.6259, d.f. = 15; Fig. 5i, for session 1, *P* = 0.008921, *t* = 3.117, d.f. = 11.99; for session 2, *P* = 0.003970, *t* = 3.453, d.f. = 13.76; for session 3, *P* = 0.004659, *t* = 3.344, d.f. = 14.412; Fig. 5k, *P* = 0.0126, *t* = 2.858, d.f. = 14, *F* = 17.18; Fig. 5l, *P* = 0.0026, *t* = 3.558, d.f. = 16, *F* = 2.473; Fig. 5m, *P* = 0.0009, *t* = 4.064, d.f. = 16, *F* = 1.133; Extended Data Fig. 1e, *P* < 0.000001, *t* = 7.991, d.f. = 20, *F* = 2.293; Extended Data Fig. 8c, for erections, *P* = 0.2420, *t* = 1.231, d.f. = 12, *F* = 2.977; for cups, *P* = 0.8338, *t* = 0.2148, d.f. = 11, *F* = 1.701; Extended Data Fig. 9a, for different interstimulus intervals in the order from small to large of females, *P* = 0.5498, 0.9012, 0.1837, 0.3384, 0.5429, for the same order of males, *P* = 0.7127, 0.5249, 0.9468, 0.9228, 0.6364; Extended Data Fig. 9b, for females, *P* = 0.7969, *t* = 0.2619, d.f. = 15, *F* = 1.409; for males, *P* = 0.6961, *t* = 0.3970, d.f. = 18, *F* = 3.950; Extended Data Fig. 9d, for number of mounts, *P* = 0.4617, *t* = 0.7416, d.f. = 49, *F* = 1.445; for mount total duration, *P* = 0.4876, *t* = 0.6994, d.f. = 49, *F* = 1.865; for mounting start time, *P* = 0.0064, *t* = 2.850, d.f. = 49, *F* = 6.615; Extended Data Fig. 9e, for post-ejaculation freeze, *P* = 0.3365, *t* = 0.9722, d.f. = 42, *F* = 1.436; for ejaculation start time, *P* = 0.2897, *t* = 1.072, d.f. = 42, *F* = 1.118; Extended Data Fig. 9f, for intromission number, *P* = 0.8697, *t* = 0.1649, d.f. = 45, *F* = 1.531; for intromission start time, *P* = 0.0287, *t* = 2.250, d.f. = 53, *F* = 1.717; for intromission total duration, *P* = 0.0252, *t* = 2.318, d.f. = 45, *F* = 3.553; for intromission duration per bout, *P* = 0.000003, *t* = 5.324, d.f. = 45, *F* = 2.618; for inter-intromission interval, *P* = 0.0222, *t* = 2.370, d.f. = 45, *F* = 9.020; Extended Data Fig. 9g, from trial 1 to 3, *P* = 0.2473, 0.4192, 0.1055, *t* = 1.216, 0.8324, 1.723, d.f. = 12, 14, 15; Extended Data Fig. 9h, from trial 1 to 3, *P* = 0.8998, 0.7724, 0.01131, *t* = 0.1287, 0.2949, 2.886, d.f. = 12, 14, 15; Extended Data Fig. 9i, from trial 1 to 3, *P* = 0.9876, 0.2966, 0.9297, *t* = 0.01601, 1.106, 0.09282, d.f. = 9.583, 9.274, 4.890; between trial 1 and 3 of Ctrl, *P* = 0.0181, *t* = 2.492; Extended Data Fig. 10b, from trial 1 to 2, *P* = 0.3629, 0.6909, *t* = 0.9335, 0.4041, d.f. = 18, 18; Extended Data Fig. 10c, from trial 1 to 2, *P* = 0.1489, 0.9108, *t* = 1.508, 0.1136, d.f. = 18, 18; Extended Data Fig. 10d, from trial 1 to 2, *P* = 0.3465, 0.9190, *t* = 0.9682, 0.1031, d.f. = 17, 18; Extended Data Fig. 10e, from trial 1 to 2, *P* = 0.2149, 0.9918, *t* = 1.286, 0.01039, d.f. = 18, 18; Extended Data Fig. 10h, *P* = 0.1140, *t* = 1.672, d.f. = 16, *F* = 1.343; Extended Data Fig. 10i, *P* = 0.0033, *t* = 3.455, d.f. = 16, *F* = 3.218; Extended Data Fig. 10j, *P* = 0.9348, *t* = 0.08315, d.f. = 16, *F* = 4.197; Extended Data Fig. 10k, *P* = 0.0126, *t* = 2.858, d.f. = 14, *F* = 17.18; Extended Data Fig. 10l, *P* = 0.4063, *t* = 0.8563, d.f. = 14, *F* = 1.829;

### Reporting summary

Further information on research design is available in the Nature Portfolio Reporting Summary linked to this article.

## Online content

Any methods, additional references, Nature Portfolio reporting summaries, source data, extended data, supplementary information, acknowledgements, peer review information; details of author contributions and competing interests; and statements of data and code availability are available at 10.1038/s41586-024-07528-4.

## Supplementary information

Reporting Summary

Supplementary Video 1 Sexual reflexes in response to 50-Hz-vibration stimuli in male mice. Vibration stimuli (50 Hz) were delivered to the externalized glans penis of the awake male mice, after spinal transection. The glans penis was positioned in a holder while being stimulated by the vibration motor. After ‘cup’ behaviour was observed, the vibration stimulator was removed.

Supplementary Video 2 Sexual reflexes in response to optogenetic stimulation of TrkB^+^ Krause afferents in male mice. In *TrkB*^*creER*^*;Avil*^*Flpo*^*;R26*^*FSF-LSL-ReaChR*^ (tamoxifen 0.5 mg i.p. injection at P5) adult male mice, optogenetic stimulation (10 Hz, 2 ms pulse for 20 s) was applied to the glans penis of the awake spinalized animals.

Supplementary Video 3 Representative mating behaviours: sniffing, mounting, intromission and ejaculation. A receptive female mouse was introduced into the home cage of an experienced male mouse. The cage cover was replaced by a custom-made wall to allow video monitoring of the mating behaviours from the top.

## Source data

Source Data Fig. 1

Source Data Fig. 2

Source Data Fig. 4

Source Data Fig. 5

Source Data Extended Data Fig. 1

Source Data Extended Data Fig. 3

Source Data Extended Data Fig. 9

Source Data Extended Data Fig. 10

## Data Availability

Datasets may be obtained from the corresponding author on reasonable request. Source data are provided with this paper.

## Code availability

Custom scripts used in this study have been posted at GitHub (https://github.com/NeuronQi/Krause_corpuscle). Additional information is available from the corresponding author on request.
