# Publisher Correction: Krause corpuscles are genital vibrotactile sensors for sexual behaviours

**DOI:** 10.1038/s41586-024-08014-7

**Published:** 2024-09-09

**Authors:** Lijun Qi, Michael Iskols, Rachel S. Greenberg, Jia Yin Xiao, Annie Handler, Stephen D. Liberles, David D. Ginty

**Affiliations:** 1 Department of Neurobiology, Howard Hughes Medical Institute, Harvard Medical School, Boston, MA USA; 2 Department of Cell Biology, Howard Hughes Medical Institute, Harvard Medical School, Boston, MA USA

**Keywords:** Touch receptors, Sexual behaviour

Correction to: *Nature* 10.1038/s41586-024-07528-4 Published online 19 June 2024

In the version of this article initially published, the text in the “DRG neurons innervating Krause corpuscles” section, now reading, in part, “An initial survey of mouse genetic tools revealed that two alleles, *TrkB*^*creER*^ (also known as *Ntrk2*) and *Ret*^*creER*^,” had a typographical error: “*Ntrk2*” appeared as “*Ntrk1*”. The error has been corrected in the HTML and PDF versions of the article.

