# Supplementary material for: Krause corpuscles are genital vibrotactile sensors for sexual behaviours
Source: Nature. 2024 Jun 19;630(8018):926–34. doi: 10.1038/s41586-024-07528-4 (PMC11208142; doi:10.1038/s41586-024-07528-4)
Supplement: Supplementary file 1 — Reporting Summary [file 41586_2024_7528_MOESM1_ESM.pdf]

nature portfolio

Corresponding author(s): David Ginty

Last updated by author(s): Mar 26, 2024

Reporting Summary

Nature Portfolio wishes to improve the reproducibility of the work that we publish. This form provides structure for consistency and transparency in reporting. For further information on Nature Portfolio policies, see our Editorial Policies and the Editorial Policy Checklist.

Statistics

For all statistical analyses, confirm that the following items are present in the figure legend, table legend, main text, or Methods section.

n/a	Confirmed	
☐	☒	The exact sample size (*n*) for each experimental group/condition, given as a discrete number and unit of measurement
☐	☒	A statement on whether measurements were taken from distinct samples or whether the same sample was measured repeatedly
☐	☒	The statistical test(s) used AND whether they are one- or two-sided<br>*Only common tests should be described solely by name; describe more complex techniques in the Methods section.*
☒	☐	A description of all covariates tested
☐	☒	A description of any assumptions or corrections, such as tests of normality and adjustment for multiple comparisons
☐	☒	A full description of the statistical parameters including central tendency (e.g. means) or other basic estimates (e.g. regression coefficient) AND variation (e.g. standard deviation) or associated estimates of uncertainty (e.g. confidence intervals)
☐	☒	For null hypothesis testing, the test statistic (e.g. *F*, *t*, *r*) with confidence intervals, effect sizes, degrees of freedom and *P* value noted<br>*Give P values as exact values whenever suitable.*
☒	☐	For Bayesian analysis, information on the choice of priors and Markov chain Monte Carlo settings
☒	☐	For hierarchical and complex designs, identification of the appropriate level for tests and full reporting of outcomes
☒	☐	Estimates of effect sizes (e.g. Cohen's *d*, Pearson's *r*), indicating how they were calculated

Our web collection on statistics for biologists contains articles on many of the points above.

Software and code

Policy information about availability of computer code

Data collection	Histological data were acquired using Zeiss Zen software (3.8). In vivo calcium imaging data were acquired using ThorCam Software (3.7.0). In vivo electrophysiology data were acquired using Intan Technologies Recording Controller (2.07), and the measured stimuli were synchronized and measured using Matlab (version R2019a or R2021a). Sexual reflexes of male mice were video recorded by SpinView (1.1.0.43). Mouse mating behaviors were recorded using IC capture (2.5).
Data analysis	Data were analyzed using Matlab version R2019a and R2021a, ImageJ (version 1.53t), Jupyter notebook (v. 6.5.4), Boris (v. 8.20.3), GraphPad Prism (Version 10), JRCLUST (version 3.2.2). We used a previously developed pipeline to analyze MEA data (https://github.com/ajemanuel/analyzeMEA). Custom scripts used in this study are posted on Github (https://github.com/NeuronQi/Krause_corpuscle).

For manuscripts utilizing custom algorithms or software that are central to the research but not yet described in published literature, software must be made available to editors and reviewers. We strongly encourage code deposition in a community repository (e.g. GitHub). See the Nature Portfolio guidelines for submitting code & software for further information.

Data

Policy information about availability of data

All manuscripts must include a data availability statement. This statement should provide the following information, where applicable:

- Accession codes, unique identifiers, or web links for publicly available datasets
- A description of any restrictions on data availability
- For clinical datasets or third party data, please ensure that the statement adheres to our policy

The data generated in this study are available from the corresponding author upon request. Source data are provided with the paper.

Research involving human participants, their data, or biological material

Policy information about studies with human participants or human data. See also policy information about sex, gender (identity/presentation), and sexual orientation and race, ethnicity and racism.

Reporting on sex and gender	N/A
Reporting on race, ethnicity, or other socially relevant groupings	N/A
Population characteristics	N/A
Recruitment	N/A
Ethics oversight	N/A

Note that full information on the approval of the study protocol must also be provided in the manuscript.

Field-specific reporting

Please select the one below that is the best fit for your research. If you are not sure, read the appropriate sections before making your selection.

☒ Life sciences ☐ Behavioural & social sciences ☐ Ecological, evolutionary & environmental sciences

For a reference copy of the document with all sections, see nature.com/documents/nr-reporting-summary-flat.pdf

Life sciences study design

All studies must disclose on these points even when the disclosure is negative.

Sample size	No sample size calculation was performed. Sample sizes were based on previous studies from our lab and others (e.g., Neubarth et al., 2020 Science).
Data exclusions	For male sexual reflex testing, males showing excessive spontaneous erections after externalizing the glans penis were excluded. For female sexual reflex testing, females showing frequent whole-body movements that strongly disrupted the vaginal pressure measurements were excluded. During mating behavior, intromission bouts lasting less than two seconds were excluded from the analysis due to the lack of rhythmicity of movements and the possibility that penetration did not fully occur. For mating assay of naturally-cycled females, the females that did not show proestrus or estrus in 10 days were excluded.
Replication	We performed experiments with multiple animals to confirm reproducibility. All attempts at replication were successful. The number of replications is noted in the figure legends.
Randomization	Stimuli of different vibration frequencies during in vivo calcium imaging or electrophysiology were randomized. The allocation of mice of different genotypes into experimental groups was randomized.
Blinding	For histological experiments, images were collected and analyzed by investigators who were blinded to genotype whenever possible. The mating behaviors were scored by an investigator blinded to the genotype of the animals, and the genotype was only revealed after quantification and summary of the entire behavioral dataset. The experimenter was not blinded to the genotype of animals during in vivo eletrophysiological recordings, in vivo calcium imaging or sexual reflex tests, because only the animals of the desired genotypes were used in the experiments.

Reporting for specific materials, systems and methods

We require information from authors about some types of materials, experimental systems and methods used in many studies. Here, indicate whether each material, system or method listed is relevant to your study. If you are not sure if a list item applies to your research, read the appropriate section before selecting a response.

Materials & experimental systems

n/a	Involved in the study
☐	☒ Antibodies
☒	☐ Eukaryotic cell lines
☒	☐ Palaeontology and archaeology
☐	☒ Animals and other organisms
☒	☐ Clinical data
☒	☐ Dual use research of concern
☒	☐ Plants

Methods

n/a	Involved in the study
☒	☐ ChIP-seq
☒	☐ Flow cytometry
☒	☐ MRI-based neuroimaging

Antibodies

Antibodies used

chicken anti-GFP (Aves Labs, GFP-1020, 1:500),
goat anti-GFP (US Biological, G8965-01E, 1:500),
goat anti-mCherry (CedarLane, AB0040-200, 1:500),
rabbit anti-CGRP (Immunostar, 24112, 1:500),
chicken anti-NF200 (Aves Labs, NFH, 1:200),
rabbit anti-NF200 (Sigma, N4142-.2ML, 1:500),
rabbit anti-S100 (ProteinTech, 15146-1-AP, 1:200-1:500),
rat anti-TROMA-1 (DSHB, AB_531826, 1:200),
goat anti-CD31 (R&D Systems, AF3628, 1:500),
guinea pig anti-FLAG (1:500) (custom-made, Handler et al., 2023)
IB4 (Alexa 647 conjugated) (Invitrogen, I32450, 1:500).

Validation

Chicken anti-GFP (Aves Labs, GFP-1020), goat anti-GFP (US Biological,G8965-01E), goat anti-mCherry (CedarLane, AB0040-200), rabbit anti-CGRP (Immunostar, 24112), chicken anti-NF200 (Aves Labs, NFH), rabbit anti-S100 (ProteinTech, 15146-1-AP) and guinea pig anti-FLAG were validated in Handler et al., 2023 (PMID: 37725982);
Rabbit anti-NF200 (Sigma, N4142-.2ML) and IB4 (Alexa 647 conjugated) (Invitrogen, I32450) were validated in Qi et al., 2024 (PMID: 38442711);
Rat anti-TROMA-1 (DSHB, AB_531826) was validated in Emanuel et al., 2021 (PMID: 34789880);
goat anti-CD31 (R&D Systems, AF3628) were validated in Ben-Zvi et al., 2014 (PMID: 24828040).

Animals and other research organisms

Policy information about studies involving animals; ARRIVE guidelines recommended for reporting animal research, and Sex and Gender in Research

Laboratory animals

All mice used in this study have been previously described, including: TrkBCreER (JAX 027214), RetCreER (MGI 4437245), AdvillinCre (JAX 032536), AdvillinFlpO (Choi et al., Nature, 2020), RetCFP (MGI 3777555), TrkBflox(Liu et al,. Science, 2012), Brn3acKOAP (JAX 010558), TauFSF-iAP (Lehnert et al., Cell, 2021), MrgprdGFP (Zylka et al., Neuron, 2005), Th2A-CreER (RRID:IMSR_JAX:025614), Mrgprb4Cre (Vrontou et al., Nature, 2013), PLPEGFP (JAX 033357), Piezo2smFP-FLAG (Handler et al., Neuron, 2023), R26FSF-LSL-ReaChR-mCitrine (JAX 024846), Ai80 (R26FSF-LSL-CatCh; JAX 025109), Ai14 (R26LSL-tdTomato; JAX 007914), Ai65 (R26FSF-LSL-tdTomato; JAX 021875), Ai96 (R26LSL-GCaMP6s; Jax 028866), and Ai148 (TIGRELSL-GCaMP6f-tTA2, 030328). All lines were kept on a mixed background, while AdvillinCre and TrkBflox were bred from mixed background to C57Bl/6 background for two generations for mating behavior testing.

Wild animals

No wild animals were used.

Reporting on sex

Both male and female mice were used for anatomical, physiological and behavioral experiments in this study.

Field-collected samples

No field-collected samples were used.

Ethics oversight

Animals were handled according to protocols approved by the Harvard Standing Committee on Animal Care following the NIH Guide for the Care and Use of Laboratory Animals. Mice were housed in a temperature-controlled and humidity-controlled facility, maintained on a 12h light/dark cycle, and given food and water ad libitum.

Note that full information on the approval of the study protocol must also be provided in the manuscript.
